# New theropod dinosaur from the Lower Cretaceous of Japan provides critical implications for the early evolution of ornithomimosaurs

**DOI:** 10.1038/s41598-023-40804-3

**Published:** 2023-09-07

**Authors:** Soki Hattori, Masateru Shibata, Soichiro Kawabe, Takuya Imai, Hiroshi Nishi, Yoichi Azuma

**Affiliations:** 1 Institute of Dinosaur Research, Fukui Prefectural University, 4-1-1 Matsuoka-kenjojima, Eiheiji, Fukui 910-1195 Japan; 2 Fukui Prefectural Dinosaur Museum, 51-11, Terao, Muroko, Katsuyama, Fukui 911-8601 Japan

**Keywords:** Palaeontology, Taxonomy

## Abstract

Ornithomimosauria consists of the ostrich-mimic dinosaurs, most of which showing cursorial adaptations, that often exhibit features indicative of herbivory. Recent discoveries have greatly improved our knowledge of their evolutionary history, including the divergence into Ornithomimidae and Deinocheiridae in the Early Cretaceous, but the early part of their history remains obscured because their fossil remains are scarce in the Aptian–Albian sediments. In recent years, many isolated ornithomimosaur remains have been recovered from the Aptian Kitadani Formation of Fukui, central Japan. These remains represent multiple individuals that share some morphological features common to them but unknown in other ornithomimosaurs, suggesting a monospecific accumulation of a new taxon. As a result of the description and phylogenetic analysis, the Kitadani ornithomimosaur is recovered as a new genus and species *Tyrannomimus fukuiensis*, the earliest definitive deinocheirid that complements our knowledge to understand the early evolutionary history of Ornithomimosauria. Due to its osteological similarity to *Tyrannomimus*, a taxon previously considered an early tyrannosauroid based on fragmentary specimens, namely *Aviatyrannis jurassica*, may represent the earliest ornithomimosaur from the Upper Jurassic of Europe, significantly expanding the temporal and biogeographic range of Ornithomimosauria. This finding fills a 20-million-year ghost lineage of Ornithomimosauria implied by the presence of the oldest fossil record of Maniraptora from the Middle Jurassic and is consistent with the hypothesis that their biogeographic range was widespread before the Pangaean breakup in the Kimmeridgian.

## Introduction

The clade Ornithomimosauria consists of ostrich-mimic dinosaurs such as *Ornithomimus*, *Struthiomimus* and *Gallimimus* that are usually interpreted as cursorial mainly based on their slender hindlimbs^1^. In addition, ornithomimosaurs often exhibit a small head with toothless jaws, rostrally-downturned mandibles and ventrally-displaced mandibular joints, and elongated neck indicating their herbivorous ecomorphology^2–4^. Recent findings on the phylogenetic position of several enigmatic taxa such as *Nqwebasaurus* and *Deinocheirus* within Ornithomimosauria have greatly improved our knowledge on their evolutionary history including the divergence to two major clades, Ornithomimidae and Deinocheiridae^5, 6^. Notably, the former exhibits adaptations for the cursoriality including the presence of the arctometatarsalian pes, which is absent in the latter. These two clades are hypothesized to have diverged earlier in the Early Cretaceous due to the inferred deinocheirid affinity of *Beishanlong* and/or *Harpymimus*^7–9^. However, other deinocheirids and ornithomimids are known mostly from the Upper Cretaceous^1^. Furthermore, remains of basal ornithomimosaurs from the upper Lower Cretaceous (Aptian–Albian) are scarce^10^, leaving the early part of the evolutionary history of ornithomimosaurs obscure.

**Figure 1:**
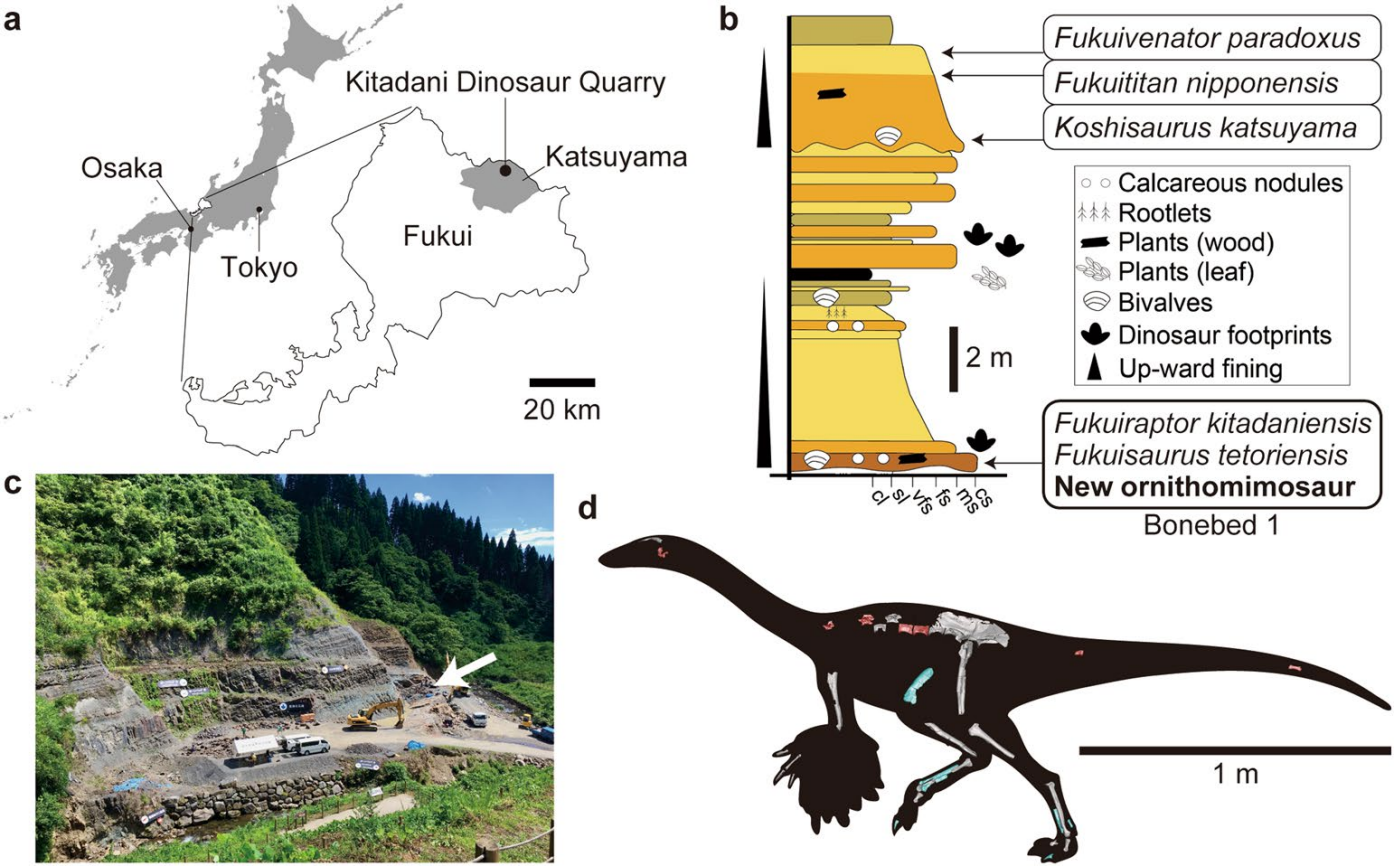
Locality, horizon, and overview of ornithomimosaur materials. Regional map for the location of Fukui in Japan, and the Kitadani Dinosaur Quarry in Fukui (**a**), stratigraphic section of the part of the Kitadani Formation in the Kitadani Dinosaur Quarry (**b**), photograph of the Kitadani Dinosaur Quarry in 2019 with an arrowhead indicating where the studied specimens were yielded (**c**), and overview of the ornithomimosaur materials (**d**). The map in (**a**) is modified from the one available at www.freemap.jp. Fossil specimens shown in (**d**) are not in the same scale while the bar is scaled for the paratype (FPDM-V-10295). The holotype (FPDM-V-11333) and paratype in (**d**) are colored in red anb blue, respectively. Right femur in (**d**) is mirrored from the left one (FPDM-V-11338).

The Aptian sediments rich in dinosaur remains occurs in Fukui, central Japan, namely the Kitadani Formation of the Tetori Group, from which three theropods, two ornithopods and a sauropod have been described as new taxa (Fig. 1). In recent years, many isolated ornithomimosaurian materials have been found in the Kitadani Dinosaur Quarry bearing the Kitadani Formation. The remains occur in the Bonebed 1 layer of the quarry and represent multiple individuals exhibiting some morphological features common among them but unknown in other ornithomimosaurs. Therefore, the ornithomimosaurian materials from the Bonebed 1 can be regarded as monospecific (see Discussion for detail) and may represent a new taxon. This study investigates the skeletal morphology and the taxonomic and phylogenetic positions of the Kitadani ornithomimosaur to provide critical insights about the Aptian-Albian ornithomimosaurs to complement our knowledge to understand the early evolutionary history of Ornithomimosauria.

## Systematic paleontology

Dinosauria Owen, 1842.

Theropoda Marsh, 1881.

Tetanurae Gauthier, 1986.

Coelurosauria von Huene, 1920.

Ornithomimosauria Barsbold, 1976.

*Tyrannomimus fukuiensis* gen. et sp. nov.

### Etymology

The genus name is derived from its morphological resemblance with tyrannosauroids, in which the vertical ridge on the ilium has been regarded as a synapomorphy; the specific name is derived from Fukui, the prefecture where the type and referred specimens were found.

### Diagnosis

Based on the holotype, theropod dinosaur with following autapomorphies among ornithomimosaurs: deep dorsal tympanic recess with its bottom subdivided by an anteroposteriorly-oriented lamina; expanded spherical cavities within prezygocentrodiapophyseal, centrodiapophyseal and postzygocentrodiapophyseal fossae.

Based on the referred specimens, deep anterolateral pit on proximal part of humerus can be another autapomorphy among ornithomimosaurs. However, this feature should be confirmed by additional specimens including humeri and some elements shared by the holotype.

*Tyrannomimus* can be distinguished from penecontemporary Asian ornithomimosaurs by having the following features: from *Harpymimus* by having the deltopectoral crest shorter than the quarter of the humeral length, the muscle scar on the anterolateral margin of the deltopectoral crest, low and elongated ilium, and concave ventral margin of the postacetabular process; from *Shenzhousaurus* by having ventral grooves on anterior caudal centra, straighter manual unguals, and the lateral distal condyle of the femur extended further distally than the medial condyle that is flattened distally.

### Holotype

FPDM-V-11311, a disarticulated but associated skeleton including two parts of the braincase, several dorsal, sacral and caudal vertebrae, and fragments of ilium.

### Paratype

FPDM-V-10295, a disarticulated but associated hindlimb skeleton including a partial left femur, left metatarsal II, left and right metatarsal IV, right pedal phalanx I-1 and a pedal ungual.

### Referred specimens

There are multiple isolated specimens including a frontal (FPDM-V-11313), 3 dorsal (FPDM-V-11314–11316) and 4 sacral (FPDM-V-11317–11320) vertebrae, 3 humeri (FPDM-V-11321–11323), 10 manual phalanges including unguals (FPDM-V-8579, 8580, 8945, 11324–11329, 11332), 6 ilia (FPDM-V-9348, 11333–11337), a femur (FPDM-V-11338), 3 tibiae (FPDM-V-11339–11341), 3 metatarsals (FPDM-V-11343–11345), and 4 pedal phalanges (FPDM-V-11343, 11346–11348). Each specimen was collected from the same layer (Bonebed I) except for FPDM-V-11343 that includes two metatarsals and one pedal phalanx. Vertebrae and ilia are shared with the holotype while the hindlimb elements are shared with the paratype. Frontal and humeri are not shared with the type specimens.

### Locality and horizon

The type and referred specimens were found from the Bonebed 1 of the Kitadani Dinosaur Quarry^11^ in the northern part of Katsuyama, Fukui. The Lower Cretaceous (Aptian^12, 13^) Kitadani Formation of the Tetori Group crops out in this quarry.

### Description

#### Frontal

**Figure 2:**
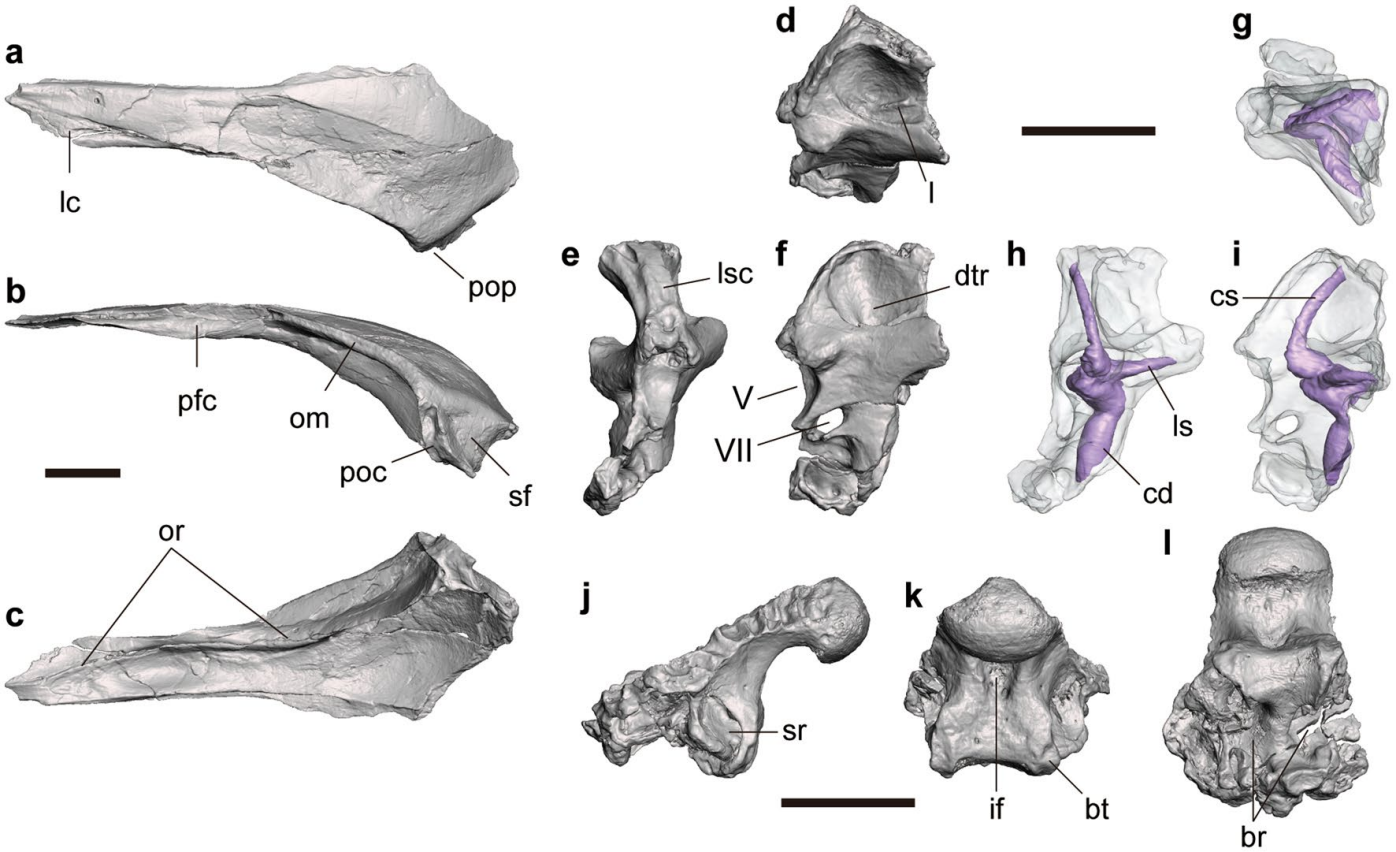
Cranial elements of *Tyrannomimus fukuiensis*. Left frontal of FPDM-V-11313 (**a**–**c**) and right prootic (mirrored; **d**–**f**), osseous labyrinth (mirrored; **g**–**i**) and basioccipital (**j**–**l**) of FPDM-V-11311 in dorsal (**a**, **g**), lateral (**b**, **f**, **i**, **j**), ventral (**c**, **l**), dorsolateral (**d**), anterior (**e**, **h**) and posterior (**k**) views. Abbreviations: *br* basisphenoid recess, *bt* basal tuber, *cd* cochlear duct, *dtr* dorsal tympanic recess, *if* infracondylar fossa, *lc* lacrimal contact, *ls* lateral semicircular canal, *lsc* laterosphenoid contact, *om* orbital margin, *or* orbitonasal ridge, *pfc* prefrontal contact, *poc* postorbital contact, *pop* postorbital process, *rs* rostral semicircular canal, *sf* supratemporal fossa, *sr* subcondylar recess, *V* trigeminal nerve opening, *VII* facial nerve opening. Scale bars equal to 10 mm.

FPDM-V-11313 is an almost complete left frontal lacking its posteromedial end (Fig. 2a–c). In dorsal view, the frontal is far longer than wide, pointed anteriorly and becomes broader posteriorly, that would form a sharp wedge between the nasals when the left and right elements are conjoined as in *Harpymimus* and more derived ornithomimosaurs^8^. In lateral view, the frontal is curved posteroventrally to form a domed structure seen in other ornithomimosaurs^1^. The anterior half of the lateral margin bears an articular surface for the lacrimal and prefrontal. The surface is flat and faces dorsally in the anterior half, while it becomes concave like a notch and faces laterally in the posterior half, which probably correspond to the facets for lacrimal and prefrontal, respectively. The supratemporal fossa occupies only the posterolateral end of the dorsal surface. This fossa is demarcated by a straight anterolaterally-directed margin which reaches the apex of the postorbital contact.

In dorsal view, the lateral margin posterior to the orbital margin smoothly transits into the postorbital process, which can be recognized by the lateralmost peak bearing a narrow flat margin facing laterally. In lateral view, this postorbital process bears a distinct groove directed ventrally for the contact with the postorbital known as a synapomorphy of Ornithomimosauria but also known in some maniraptorans^5, 8^. In ventral view, the orbitonasal ridge emerges at the anterior end of the bone as a low ridge and then forms an arch reaching the lateral margin occupying the anterior two thirds. At the peak of the arch, the ridge merges with the ventral protrusion of the prefrontal contact to form a thick rounded crest. The crest becomes thicker as a deep lamina in the posterior third curving laterally to reach the ventral margin of the postorbital contact.

#### Braincase

FPDM-V-11311 (holotype) includes a right prootic (Fig. 2d–f) and a basioccipital (Fig. 2j–l). The anterior half of the osseous labyrinth is preserved inside the prootic, whose endocast has been reconstructed virtually (Fig. 2g–i).

On the prootic, the dorsal part of the lateral aspect is excavated medioventrally by a large fossa, namely the dorsal tympanic recess or the prootic pneumatic recess (Fig. 2f). The anterodorsal rim of this recess is flared laterally to bear an articular facet for the laterosphenoid facing anterodorsally. In addition, the ventral end of the dorsal tympanic recess is laterally covered by a lamina. However, the dorsal tympanic recess is not enclosed posteriorly within the prootic so that it is expanded onto the exoccipital-opisthotic (otoccipital). The ventral bottom of the dorsal tympanic recess is divided mediolaterally by another lamina, so that there are two anteroposteriorly-oriented grooves (Fig. 2d). At the level with the ventral bottom of the dorsal tympanic recess, a blunt process is present on the anterolateral margin (Fig. 2f). On the anterior margin, the laterosphenoid contact faces anterodorsally in the proximal one third (Fig. 2e). The middle one third of the anterior margin bears a spherical concavity forming the posterior rim of the exit for maxillary and mandibular branches of trigeminal nerve.

Based on the partial virtual endocast that exhibits the anterior parts of the rostral and lateral semicircular canals and the cochlear duct, the overall morphology and the length (7.88 mm) of the cochlear duct of *Tyrannomimus* are similar to those of *Struthiomimus* (AMNH FR 5355: ca. 7 mm). The length of the cochlear duct generally relates to the hearing frequency^14^. Therefore, it is suggested that the audibility of *Tyrannomimus* was comparable to that of *Struthiomimus*.

The basioccipital forms most of the occipital condyle and ventral margin of the foramen magnum. The neck of the occipital condyle is constricted in height by a ventral excavation but not in width (Fig. 2j, l). The ventral aspect of the neck is further excavated by a single, narrow median fossa representing the infracondylar fossa that also excavates the posterior surface below the condyle (Fig. 2k). The infracondylar fossa is also seen in *Garudimimus*^15^ but not in *Gallimimus* and *Ornithomimus*^5^. The ventral half of the posterior surface is shallowly concave and laterally demarcated by the basal tubera at the level with the lateral margins of the occipital condyle. Each of the basal tubera is elongated dorsoventrally and bears a shallow longitudinal groove. Dorsolateral to the basal tuber, there is a large pneumatic fossa representing the subcondylar recess on each side (Fig. 2j). The deepest part of the subcondylar recess is situated at its posterior margin and has a slit for the basisphenoid recess. The subcondylar recess connected with the internal chamber is known as a diagnostic feature of ornithomimids and tyrannosaurids^16–20^. The basisphenoid recess within the basioccipital is well developed and its ventral opening is divided laterally by a septum (Fig. 2l). Both parts of the recess invade the bone posterodorsally to reach the occipital condyle.

#### Dorsal vertebrae

**Figure 3:**
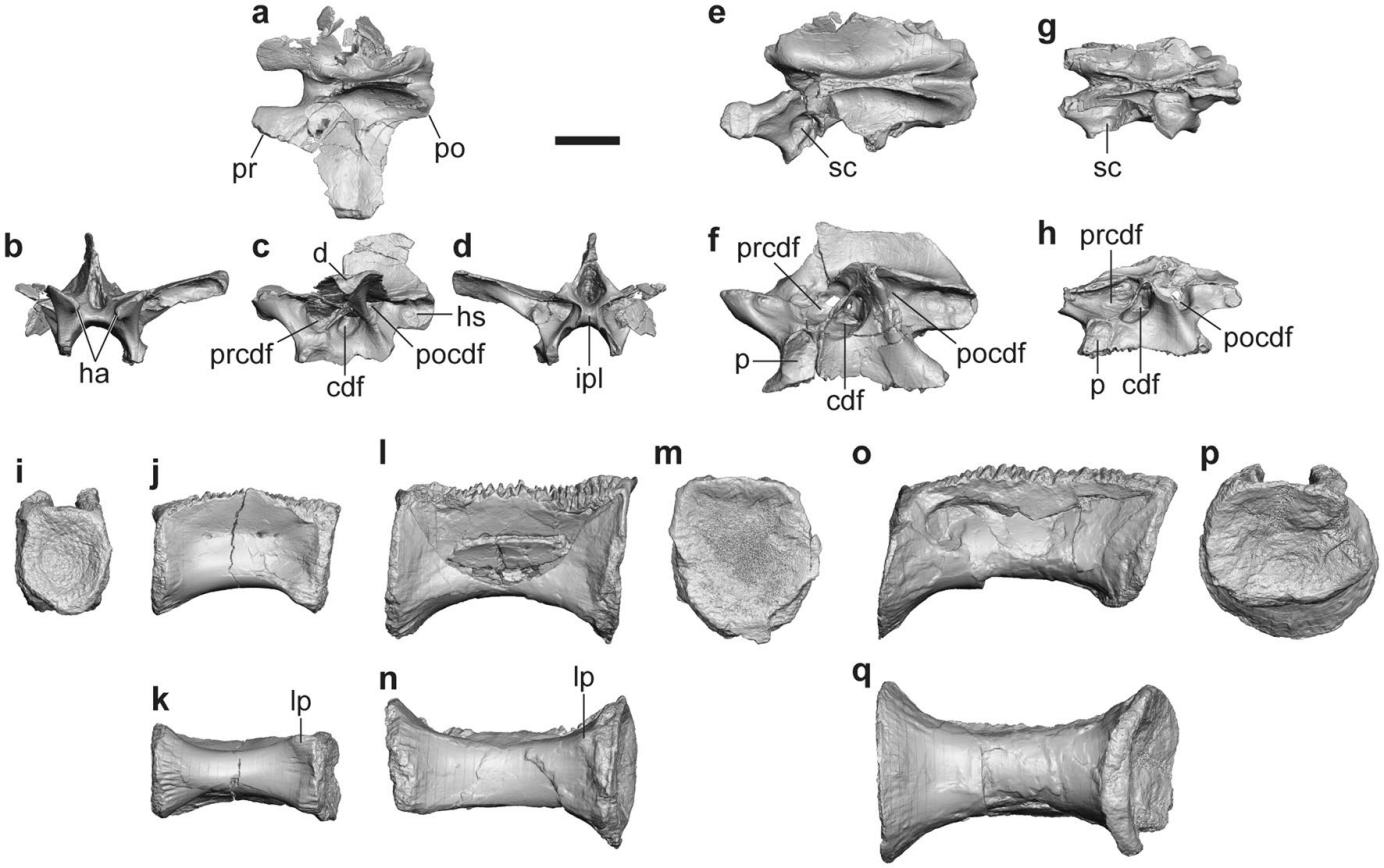
Dorsal vertebrae of *Tyrannomimus fukuiensis*. Anterior dorsal neural arch (FPDM-V-11311; **a**–**d**), middle dorsal neural arches of FPDM-V-11311 (mirrored; **e**, **f**) and FPDM-V-11314 (mirrored; **g**, **h**), and middle (FDPM-V-11315; **i**–**k**), posterior (FPDM-V-11311; **l**–**n**) and posteriormost (FDPM-V-11316; **o**–**q**) dorsal centra in dorsal (**a**, **e**, **g**), anterior (**b**, **i**), lateral (**c**, **f**, **h**, **j**, **l**, **o**), ventral (**k**, **n**, **q**) and posterior (**m**, **p**) views. Abbreviations: *cdf* centrodiapophyseal fossa, *d* diapophysis, *ha* hypantrum, *hs* hyposphene, *ipl* intrapostzygapophyseal lamina, *lp* lateroventral prominence, *p* parapophysis, *po* postzygapophysis, *pocdf* postzygocentrodiapophyseal fossa, *pr* prezygapophysis, *prcdf* prezygocentrodiapophyseal fossa, *sc* spherical cavity within prezygocentrodiapophyseal fossa. Scale bar equals to 10 mm.

FPDM-V-11311 (holotype) includes anterior and middle neural arches and two posterior centra (Fig. 3a–f, l–m). FPDM-V-11314 is a middle neural arch of a smaller individual (Fig. 3g, h). FPDM-V-11315 and 11316 represent nearly complete centra of the middle and posteriormost dorsal vertebrae, respectively (Fig. 3i–k, o–q).

In the dorsal neural arches, the postzygapophyses abut one another to form a thick lamina by fusing opposite hyposphenes (Fig. 3a, d, e, g). This condition is known as a synapomorphy of Ornithomimosauria^8^, although it is observed at least in a therizinosaurid *Alxasaurus*, an oviraptorid *Avimimus* and an alvarezsaur *Patagonykus*^5^. In the anterior dorsal neural arches, the neural spine is dorsoventrally shallow and anteroposteriorly narrow, and the transverse processes are anteroposteriorly broad, as in other coelurosaurs (Fig. 3a, c). Below the transverse process plane, the prezygocentrodiapophyseal, centrodiapophyseal and postzygocentrodiapophyseal fossae are deeply excavated and swollen to form a spherical cavity for each (Fig. 3c, e–h).

Each dorsal centrum is anteroposteriorly elongated, much longer than high (Fig. 3j, l, o) as in other ornithomimosaurs except for *Garudimimus* and *Deinocheirus*, as well as in some theropods^8^. Both the anterior and posterior articular surfaces are shallowly concave (platycoelous condition) and exhibit subcircular profiles (Fig. 3i, m, p). The ventral surface is smoothly convex at least in the anterior part of the middle centrum and the whole of the posteriormost centrum, whereas the posterior part of the middle centrum and the whole of the posterior centrum exhibit flat ventral surfaces (Fig. 3k, n, q). In the middle and posterior centra, the posterior end of the ventral surface is slightly concave due to a pair of small lateroventral prominences, a condition also known in the fifth and sixth dorsal centra of *Garudimimus* and the sixth to eighth dorsal centra of *Ornithomimus*^15^. In lateral view of the middle to posterior dorsal vertebrae, when the anterior and posterior surfaces are oriented vertically, the former is located slightly more ventrally than the latter (Fig. 3k, n) as in those of *Garudimimus* (Figs. 7E and 8B, C, E of Kobayashi and Barsbold^15^) and the posterior dorsal vertebrae of *Gallimimus* (Fig. 9E of Watanabe et al.^21^). The lateral surface is crushed in all of four centra probably due to the presence of the internal pneumatic cavity possibly connected with several small foramina on the surface (Fig. 3j, l, o). In lateral view, the ventral margin of the middle and posterior centra is substantially concave whereas the anterior one is much shallower.

#### Sacral vertebrae

**Figure 4:**
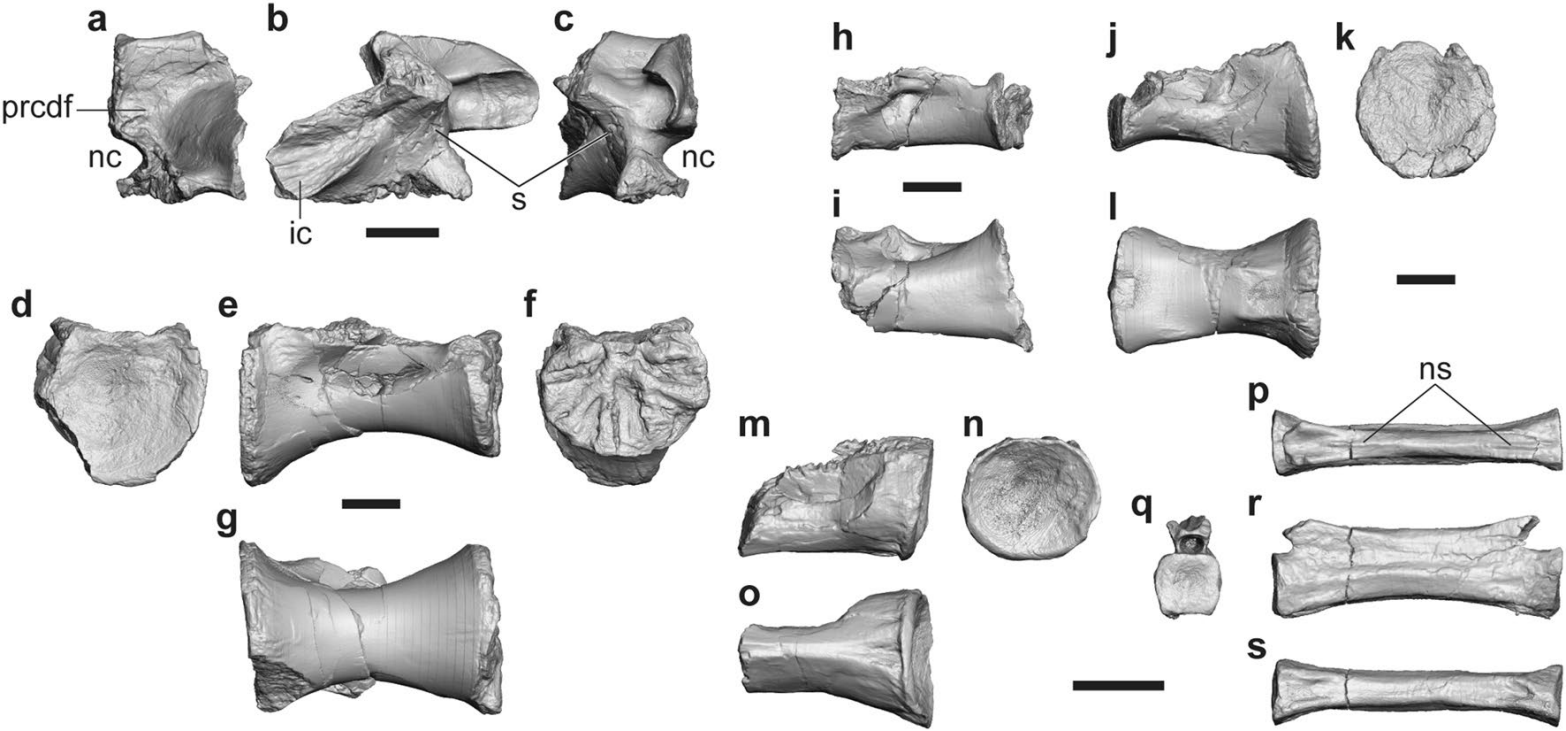
Sacral and caudal vertebrae of *Tyrannomimus fukuiensis*. Partial fifth sacral neural arch (FPDM-V-11311, mirrored; **a**–**c**), first (FPDM-V-11317; **d**–**g**), third (FPDM-V-11319; **h**, **i**) and fourth (FPDM-V-11320; **j**–**l**) sacral centra, and anterior caudal centrum (mirrored; **m**–**o**) and posterior caudal vertebra (**p**–**s**) of FPDM-V-11311 in anterior (**a**, **d**, **q**), lateral (**b**, **e**, **h**, **j**, **m**, **r**), posterior (**c**, **f**, **k**, **n**) and ventral (**g**, **i**, **l**, **o**, **s**) views. Abbreviations: *ic* iliac contact, *nc* neural canal, *ns* neural spine, *s* suture between rib and neural arch. Scale bars equal to 10 mm.

A partial neural arch and four sacral centra are described here. FPDM-V-11311 (holotype) includes a partial right neurapophysis of the posteriormost, tentatively regarded as the fifth, sacral vertebra (Fig. 4a–c). The first sacral centra is represented by FPDM-V-11317 and 11318. FPDM-V-11319 and 11320 are middle sacral centra probably representing the third and fourth, respectively (Fig. 4d–l).

In the sacral neural arch, the postzygapophyseal facet is rounded to form a single, anteroposteriorly-elongated concavity with the hyposphene facet (Fig. 4b, c). The sacral rib is fused to the neurapophysis, whereas the suture between them is partially visible as an indented line. This suture line appears lateral to the anterior end of the postzygapophysis and runs anteroventrally to meet another, horizontal suture for the centrum. The rib exhibits the ventral part of the iliac contact whereas the dorsal part is not preserved. The broad dorsomedial surface above the ventral iliac contact representing an unusually deep fossa is confluent with the prezygocentrodiapophyseal fossa excavating the base of neural arch posteriorly (Fig. 4a).

Similar to the posteriormost dorsal centrum, each sacral centrum is unusually elongated anteroposteriorly and constricted mediolaterally at its middle part to exhibit a spool-shaped profile (Fig. 4g, i, l), as in other ornithomimosaurs (e.g. Fig. 9A of Osmólska^22^; Fig. 3D of Smith^23^). In lateral view, the middle part is also constricted dorsoventrally to exhibit a spool-shaped profile in the first centrum (Fig. 4e), while this feature is absent or only slightly present in the middle centra (Fig. 4h, j). As in dorsal centra, the middle part of each sacral centrum is crushed, indicating that there was a hollow cavity inside. While the anterior articular surface of the first centrum is mediolaterally wide as dorsoventrally high (Fig. 4d), it is wider than high in the posterior surface (Fig. 4f). Such low height seems to be retained until the anterior part of the fourth centrum, whereas the articular surfaces among them are not well preserved. The posterior articular surface of the fourth centrum recovers the height equaling its width (Fig. 4k). The articular surfaces among the first and third sacral centra have grooves extending radially from the center of each dorsal margin as the sutural facets, whereas the posterior surface of the fourth exhibits a smooth, slightly concave facet probably for the unfused fifth centrum. The ventral surface is rounded in the first centrum but becomes flatter in the third (Fig. 4 g, i). The fourth centrum exhibits a characteristic ventral surface, which is concave at the anterior margin, flat in the anterior-middle part, and then forms a shallow longitudinal groove demarcated laterally by distinct ridges toward the posterior end (Fig. 4l).

#### Caudal vertebrae

FPDM-V-11311 (holotype) includes an anterior caudal centrum (Fig. 4m–o) and a posterior caudal vertebra (Fig. 4p–s), respectively. The posterior articular surface of the anterior caudal centrum is concave with oval profile compressed slightly dorsoventrally. The middle part of the caudal centrum is constricted mediolaterally to bear a narrow ventral surface occupied by a longitudinal groove. Although the pre- and postzygapophyses are not preserved, the posterior caudal vertebra is far elongated compared to the dorsoventral height and mediolateral width. The neural spine is present as a low ridge that disappears at the midlength so that it is divided into the anterior and posterior alae.

#### Humerus

**Figure 5:**
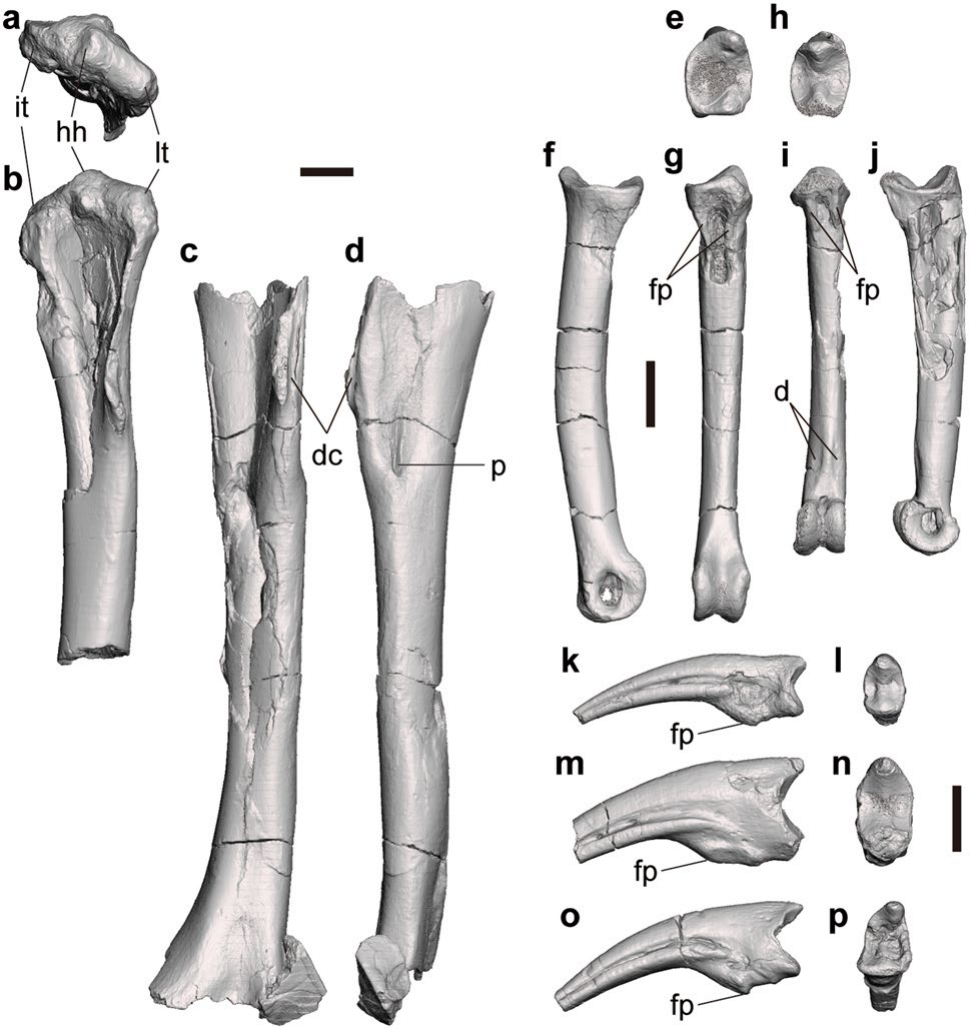
Forelimb elements of *Tyrannomimus fukuiensis*. Right humerus (FPDM-V-11321, mirrored; **a**, **b**), left humerus (FPDM-V-11322; **c**, **d**), right manual phalanx I-1 (FPDM-V-8945, mirrored; **e**–**g**), left manual phalanx II-2 (FPDM-V-11327; **h**–**j**) and manual unguals FPDM-V-8579 (**k**, **l**), 8580 (**m**, **n**) and 11332 (**o**, **p**) in proximal (**a**, **e**, **h**, **l**, **n**, **p**), anterior (**b**, **c**), lateral (**d**, **f**, **k**, **m**, **o**), palmar (**g**, **i**) and medial (**j**) views. Abbreviations: *d* depression, *dc* m. deltoideus clavicularis insertion scar on deltopectoral crest, *fp* flexor process, *hh* humeral head, *it* interal tuberosity, *lt* lateral tuberosity, *p* pit. Scale bars equal to 10 mm.

There are three specimens representing the humerus of *Tyrannomimus*. FPDM-V-11321 preserves about 10 cm from the proximal end of a right humerus (Fig. 5a, b). FPDM-V-11322 is the most complete among the three and represents a left humerus lacking both proximal and distal ends (Fig. 5c, d). FPDM-V-11323 is a much smaller specimen showing the proximal half with nearly complete proximal end.

The humerus is nearly straight in lateral view rather than being sigmoidally curved as in most theropods (Fig. 5d). However, the bone slightly curves distomedially in anterior view like most theropods (Fig. 5c). The lateral tuberosity and the internal (medial) tuberosity are rounded and confluent with the humeral head (Fig. 5b). In addition, both tuberosities are located slightly lower than the humeral head to form a triangular profile in anterior view. The internal tuberosity (or bicipital crest) is small as in other ornithomimosaurs^1^. The deltopectoral crest is small and situated proximally as in other ornithomimosaurs^1^ except for *Nqwebasaurus* (Fig. 2 of de Klerk et al.^24^). The anterior margin of the crest bears a longitudinal groove probably for the insertion of m. deltoides clavicularis. At the level with the distal end of the deltopectoral crest, a deep pit excavates the anterolateral surface distomedially with accompanying a groove extending proximally (Fig. 5d).

#### Manual phalanges

Five penultimate phalanges and four unguals are described here. FPDM-V-11324 and 11325 are the left phalanx 1 of digit I (I-1) whereas FPDM-V-8945 is the right phalanx I-1 (Fig. 5e–g). FPDM-V-11327 and 11328 are the left phalanx II-2 (Fig. 5h–j). Manual unguals are represented by FPDM-V-8579, 8580, 11329 and 11332 (Fig. 5k–p).

As in other ornithomimosaurs, manual phalanges I-1 and II-2 are strikingly elongated to form slender digits. In proximal view, the articular surfaces are deeper dorsoventrally than wide mediolaterally (Fig. 5e, h). The dorsal and ventral margins of the surface are convex and concave, respectively. There is a dorsoventral ridge dividing the surface mediolaterally. This ridge is located somewhat medially in I-1, whereas it is present just on the midline in II-2. The proximal end of the ventral surface bears a concavity demarcated laterally by a pair of longitudinal ridges (Fig. 5g, i) that correspond to the flexor processes characteristic to ornithomimosaurs^25^. Distal to this concavity, the ventral surface of the diaphysis is flat to slightly convex. In II-2, the ventral surface next proximal to the distal condyles bears a pair of rugose depression on the medial and lateral margins, which probably represents the attachment surface for soft tissues such as tendons or ligaments. Distal condyles are expanded more ventrally than dorsally in lateral view (Fig. 5f, j). A pair of collateral ligament fossae excavates the area slightly dorsal to the midline on both the medial and lateral aspects of the condyles.

On the manual ungual, the flexor tubercle is relatively small and located somewhat distally from the proximal end (Fig. 5k, m, o) as in other ornithomimosaurs^1^. The curvature is weak in lateral views of all specimens and similar to those of the second and third digits of most ornithomimosaurs^26–29^; nonetheless, it is straighter than those of *Shenzhousaurus*^30^ and *Sinornithomimus*^31^.

#### Ilium

**Figure 6:**
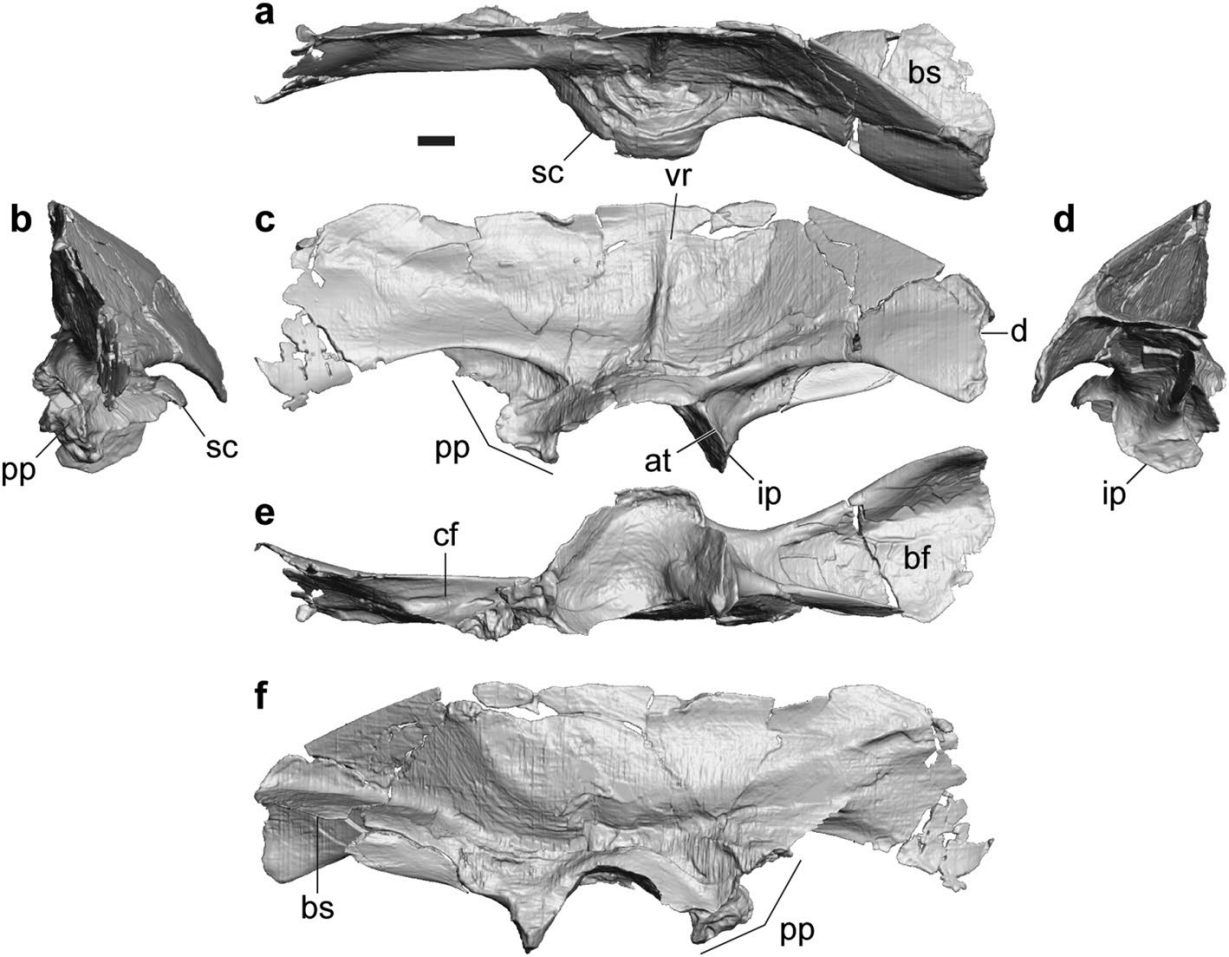
Ilium of *Tyrannomimus fukuiensis*. Left ilium (FPDM-V-11333) in dorsal (**a**), anterior (**b**), lateral (**c**), posterior (**d**), ventral (**e**) and medial (**f**) views. Abbreviations: *at* antitrochanter, *bf* brevis fossa, *bs* brevis shelf, *cf* cuppedicus fossa, *d* depression, *ip* ischial peduncle, *pp* pubic peduncle, *vr* vertical ridge, *sc* supraacetabular crest. Scale bar equals to 10 mm.

FPDM-V-11333 represents a nearly complete left ilium (Fig. 6). In addition, there are six partial materials with the acetabular part, namely FPDM-V-9348, 11311 (holotype), 11334–11337. Following description is mostly based on FPDM-V-11333.

The ilium is characterized by a narrow, sharply defined vertical ridge on the lateral surface of iliac blade above the acetabulum (Fig. 6c). This ridge may mark the border between the origins of two heads of m. iliofemoralis^32–34^ or m. iliofemoralis externus and m. iliofibularis^35–37^. Although this feature is known as a synapomorphy of Tyrannosauroidea seen in tyrannosaurids, *Stokesosaurus*, *Aviatyrannis*^38^ and *Guanlong*^39^, it can also be recognized in some ornithomimosaurs such as *Shenzhousaurus* (Figs. 2, 10 of Ji et al.^30^), *Harpymimus* (Fig. 6.8A of Kobayashi and Barsbold^27^) and the ornithomimid UCMP 154759^35^. In addition, the ilium is low and elongated as in *Aviatyrannis*^38^ and *Shenzhousaurus*^30^. In fact, the iliac blade is mediolaterally thin, dorsoventrally shallow, and anteroposteriorly elongated to be more than three times longer than the dorsoventral depth above the acetabulum. The length of the preacetabular process is subequal to that of the postacetabular process. The dorsoventral depth is also subequal with each other, whereas that of the postacetabular process slightly decreases posteriorly.

Although some parts are damaged, the preserved portion of the iliac blade exhibits a smoothly flat-to-convex dorsal margin in lateral view (Fig. 6c). When the acetabular facet is oriented horizontally, the dorsal margin of the anterior two-thirds forms a straight line in dorsal view (Fig. 6a), probably for the contact with the sacral neural spines or the opposite iliac blade as seen in tyrannosaurids, other ornithomimosaurs, alvarezsaurs and oviraptorosaurs^5, 40^. The preacetabular process mainly faces laterally but somewhat dorsally in the anterior and ventral margins, and the postacetabular blade faces slightly more dorsally. Such a twisted condition of the iliac blade is also seen in other ornithomimosaurs such as *Garudimimus* (Fig. 9A of Kobayashi and Barsbold^15^), *Rativates* (Fig. 6C of McFeeters et al.^41^), and *Gallimimus* (Pl. XLV Fig. 2a of Osmólska et al.^22^). In addition, the postacetabular process is oriented posterolaterally to diverge from the opposite iliac blade as in other ornithomimosaurs^42^.

Although the anterior margin and the anteroventral corner of the preacetabular process are not preserved, the preserved portion of the ventral margin exhibit a concave profile in lateral view (Fig. 6c). The posterodorsal corner of the postacetabular process is ornamented by a rugosity on the lateral surface along the margin. While the posterior margin is nearly vertical in lateral view, it exhibits a slight depression in the dorsal third. The presence of such depression is similar to the one posterior to the processus supratrochantericus in the dromaeosaurid *Unenlagia* (Fig. 7.1A of Novas^43^), although the latter is situated slightly posterior to the acetabulum whereas the one in FPDM-V-11333 is far posterior to the acetabulum.

The cuppedicus fossa occupies about the posterior half of the ventral margin of preacetabular process (Fig. 6c, e). The fossa faces laterally and extends posteriorly to the level with the anterior end of the acetabulum. The dorsal rim of the cuppedicus fossa overhangs laterally but becomes less distinct posteriorly. The medial wall of the cuppedicus fossa is formed by a flange continuing from the anterior margin of the pubic peduncle.

The pubic peduncle is dorsoventrally shallow and anteroposteriorly long (Fig. 6c, e). The contact facet for the pubis faces anteroventrally with its anterior half facing more anteriorly than the posterior half. Similar condition can be seen at least in *Garudimimus* (Fig. 9B of Kobayashi and Barsbold^15^) and LH-02-01 (Fig. 2D, E of Yao et al.^7^). Although the lateral profile of the anterior part of the facet is concave in FPDM-V-11333, it is not the case in the larger specimen FPDM-V-11336.

The supraacetabular crest arises from the dorsal part of the acetabular edge of the pubic peduncle (Fig. 6c). The crest projects well laterally to form a semicircular hood over the femoral head unlike smaller crests of derived tyrannosauroids^44^. Posterior to the acetabulum, the ischial peduncle exhibits a thin lateral flange as the antitrochanter seen in most coelurosaurs^42^. The ventral end of the relatively short ischial peduncle is tapered in lateral view, indicating the presence of peg-and-socket articulation between the ilium and ischium as in other ornithomimosaurs^1^. The ventral end of the peduncle is bluntly pointed in posterior view with a distinct facet demarcated dorsally by a horizontal ridge (Fig. 6d), in contrast to the anterior aspect without an apparent facet for the ischium.

The brevis fossa is relatively shallow and broad, and the brevis shelf projects medially and overhangs ventrally (Fig. 6d, e). Toward the posterior end, the medial and lateral margins of the brevis shelf diverge to make the shelf mediolaterally broader and terminates in a rounded margin in ventral view as in other ornithomimosaurs^7, 15, 30^ but unlike those of tyrannosauroids with subparallel margins (e.g. Fig. 20 of Osborn^45^; Fig. 9 of Mallon et al.^46^). In lateral view, the ventral margin of the postacetabular process is concave to obscure the brevis fossa near its posterior end (Fig. 6c) as in *Shenzhousaurus*^30^ but unlike straight ventral margins of other ornithomimosaurs^15, 27^.

#### Femur

**Figure 7:**
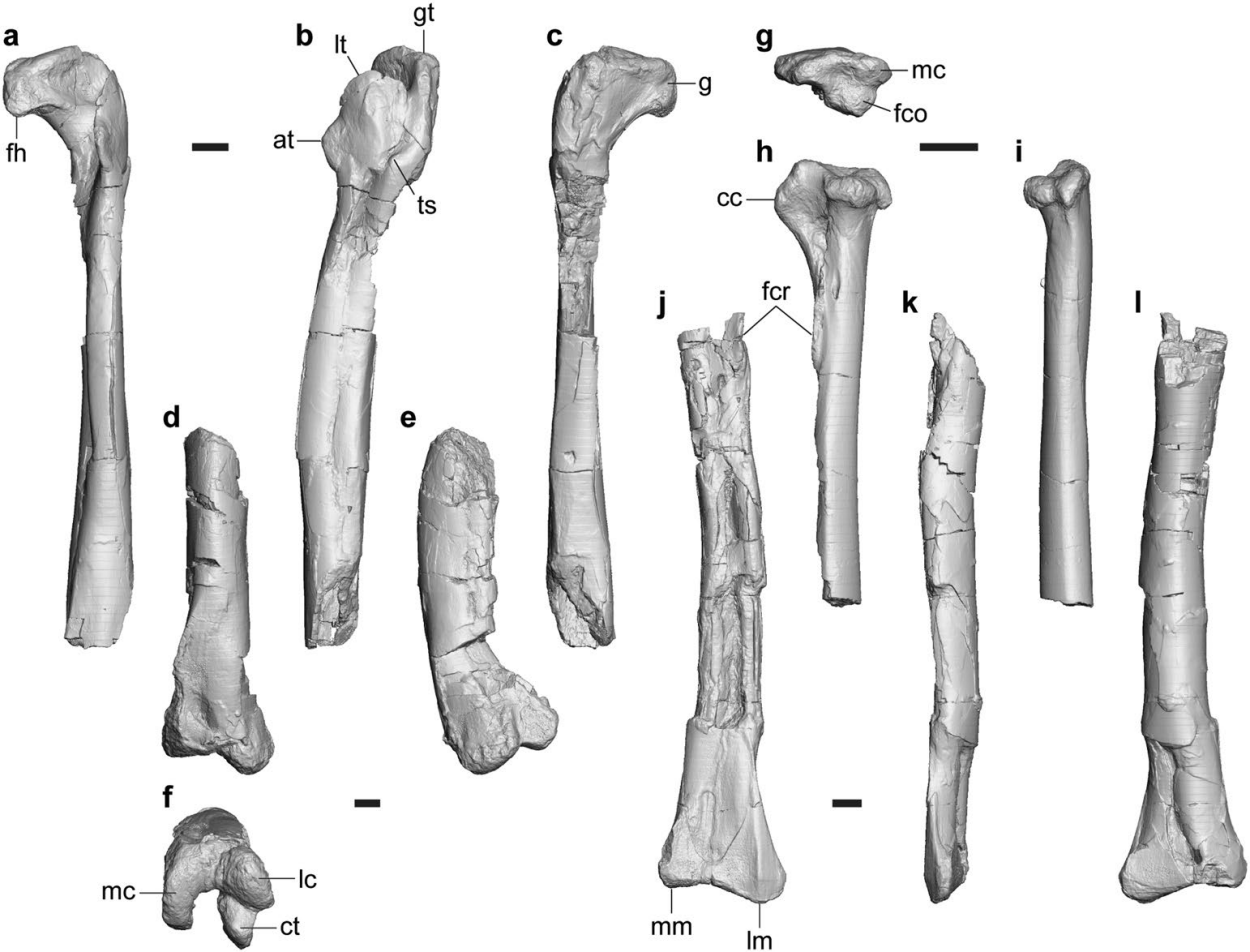
Femur and tibia of *Tyrannomimus fukuiensis*. Left femora of FPDM-V-11338 (**a**–**c**) and 10295 (**d**–**f**), right tibia (FPDM-V-11339, mirrored; **g**–**i**) and left tibia (FPDM-V-11340; **j**–**l**) in anterior (**a**, **d**, **j**), lateral (**b**, **e**, **h**, **k**), posterior (**c**, **i**, **l**), distal (**f**) and proximal (**g**) views. Abbreviations: *at* accessory trochanter, *cc* cnemial crest, *ct* crista tibiofibularis, *fco* fibular condyle, *fcr* fibular crest, *fh* femoral head, *g* groove, *gt* greater trochanther, *lc* lateral condyle, *lm* lateral malleolus, *lt* lesser trochanter, *mm* medial malleolus, *ts* trochanteric shelf. Scale bars equal to 10 mm.

FPDM-V-11338 is a left femur lacking the part around the posterior portion of the fourth trochanter and the distal end (Fig. 7a–c). FPDM-V-10295 includes the distal half of a left femur including the complete distal end (Fig. 7d–f).

The femoral head bears a roughly horizontal but slightly lateroventrally-inclined groove posteriorly (Fig. 7c). While the lesser trochanter extends dorsally on the lateral margin, it does not reach the level with the greater trochanter (Fig. 7b) as in *Garudimimus* (Fig. 13C of Kobayashi and Barsbold^15^), *Rativates*^41^ and the Bissekty ornithomimid^8^. The accessory trochanter is large and distinct form the lesser trochanter in lateral view as in *Garudimimus* (Fig. 13C of Kobayashi and Barsbold^15^) and the Bissekty ornithomimid^8^. At the level of the distal end of the accessory trochanter, the posterior half of the lateral aspect bears a distinct boss representing the trochanteric shelf. The anteromedial margin of the distal part is conspicuous as a flange expanding medially (Fig. 7d) as in *Beishanlong*^5^ and the Bissekty ornithomimid^8^.

The ventral aspect of the shaft forms a flat surface facing posteromedially to form an acute lateroventral corner with the lateral surface (Fig. 7b, c). The extensor groove on the anterior surface of the distal end, seen in derived ornithomimosaurs^8^, is absent (Fig. 7d) as in *Nqwebasaurus*^24^ and *Garudimimus* (Fig. 13F of Kobayashi and Barsbold^15^). The lateral distal condyle is extended further distally than the medial one as in *Garudimimus* (Fig. 13A of Kobayashi and Barsbold^15^) and not conical unlike those of ornithomimids^8^. There is a cleft between the lateral condyle and the crista tibiofibularis which continues to the distal surface so that it is visible in lateral view (Fig. 7e, f) as in other ornithomimosaurs^8, 15^. As in *Garudimimus*^15^, the crista tibiofibularis is pointed posteriorly and does not curve laterally in distal view.

#### Tibia

There are three slender tibiae that vary in size. FPDM-V-11339 is the smallest specimen preserving slightly over the proximal half including the proximal end (Fig. 7g–i). On the other hand, FPDM-V-11340 is the largest specimen preserving over the distal half including the distal end (Fig. 7j–l). FPDM-V-11341 is the middle-sized specimen representing the complete diaphysis but lacks the proximal end and the medial tip of the distal end.

The cnemial crest extends more proximally than the posterior condyles (Fig. 7h) as in some theropods including *Garudimimus*^15^, *Deinocheirus*^6^ and *Archaeornithomimus*^23^. In proximal view, the fibular condyle projects laterally from the posterior end of the cnemial crest, whereas it lacks the anterior process (Fig. 7g) unlike those of derived tyrannosauroids and alvarezsaurids^8^. The medial condyle is pointed posteriorly and divided from the fibular condyle by a shallow cleft. In posterior view, the medial margin is concave just below the medial condyle (Fig. 7i) as in the Angeac theropod (Fig. 2D of Allain et al.^47^). The fibular crest is low and rounded (Fig. 7h, j)as in ornithomimids^8^. The crest is clearly separated from the proximal articular surface, proximodistally short and does not reach the quarter of the length even within the diaphysis. The diaphysis is bowed medially in the middle-sized and largest specimens (Fig. 7j). There is no bracing or depression for the ascending process of the calcaneum as in other coelurosaurs^40^. The lateral malleolus extends somewhat laterally and slightly distally than the medial malleolus as in *Beishanlong*^48^ and the Bissekty ornithomimid^8^.

#### Metatarsus

**Figure 8:**
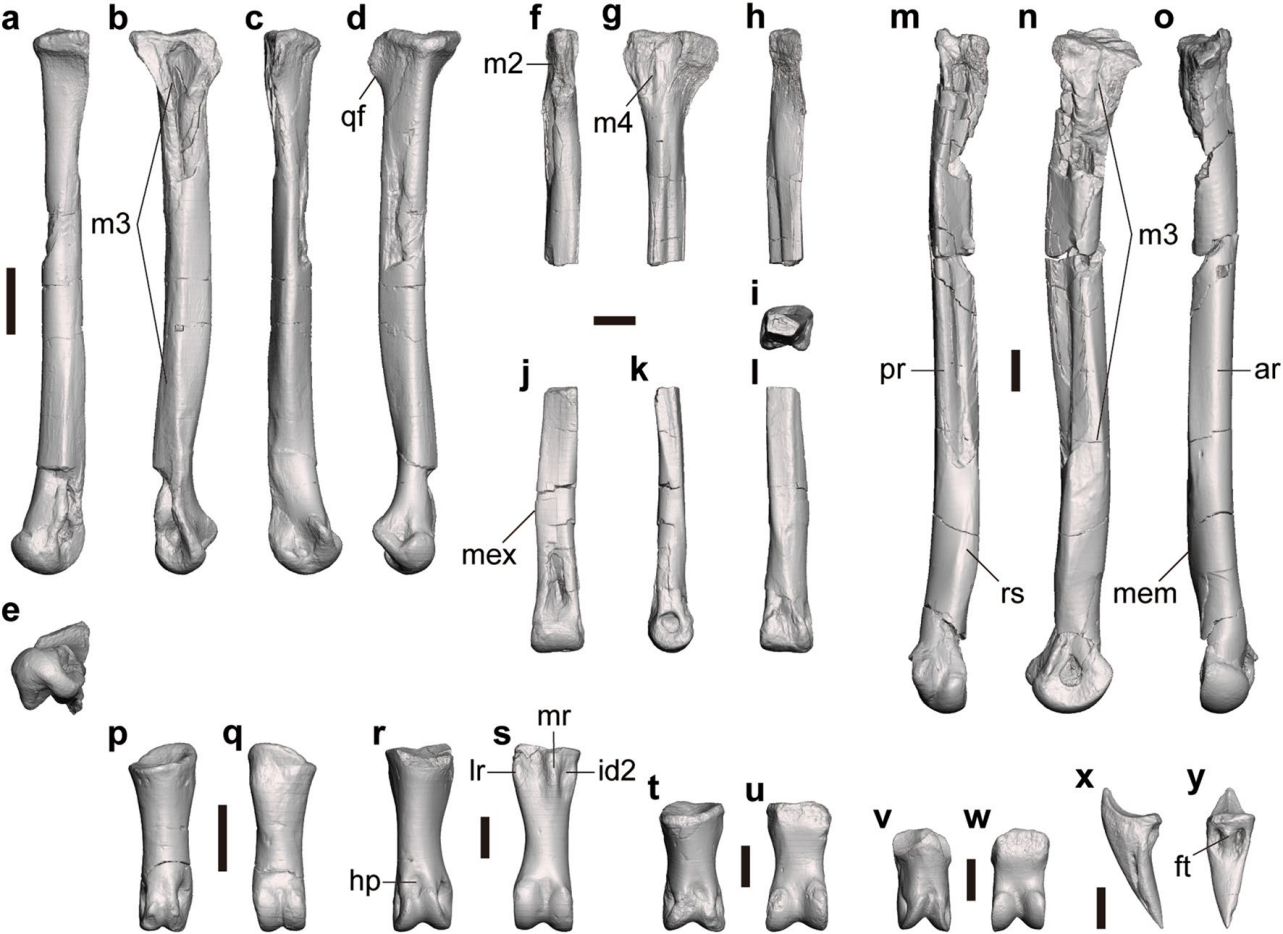
Pedal elements of *Tyrannomimus fukuiensis*. Right metatarsal II (FPDM-V-8582, mirrored; **a**–**e**), proximal part of left metatarsal III (FPDM-V-11343; **f**–**h**), distal part of left metatarsal III (FPDM-V-11344; **i**–**l**), right metatarsal IV (FPDM-V-11343, mirrored; **m**–**o**), right pedal phalanx I-1 (FPDM-V-10295, mirrored; **p**, **q**), left pedal phalanx II-1 (FPDM-V-11346; **r**, **s**), left pedal phalanx IV-1 (FPDM-V-11348; **t**, **u**), right pedal phalanx IV-2 (FPDM-V-11343; **v**, **w**) and pedal ungual (FPDM-V-10295; **x**, **y**) in anterior (**a**, **f**, **j**, **o**, **p**, **r**, **t**, **v**), lateral (**b**, **g**, **k**, **x**), posterior (**c**, **h**, **l**, **m**), medial (**d**, **n**), distal (**e**), proximal (**i**) and plantar (**q**, **s**, **u**, **w**, **y**) views. Abbreviations: *ar* anterior ridge, *ft* flexor tubercle, *hp* hyperextensor pit, *id2* insertion of m. interosseous dorsalis digiti II, *lr* lateral ridge, *m2* contact surface for metatarsal II, *m3* contact surface for metatarsal III, *m4* contact surface for metatarsal IV, *mem* medial eminence, *mex* medial expansion, *mr* medial ridge, *pr* posterior ridge, *qf* quadrangular flange, *rs* rugose surface. Scale bars equal to 10 mm.

Several metatarsals can be assigned to *Tyrannomimus fukuiensis*. FPDM-V-10295 includes the left metatarsal II lacking the proximal end and left and right metatarsal IV. FPDM-V-8582 represents a complete right metatarsal II (Fig. 8a–e). FPDM-V-11343 includes the proximal part of a left metatarsal III (Fig. 8f–h) and a right metatarsal IV (Fig. 8m–o). FPDM-V-11344 and 11345 represent the distal parts of left metatarsal III (Fig. 8i–l). There is no trace of fusion with the tarsus or among the metatarsals.

Metatarsal II has a slender diaphysis that distally diverges medially from its long axis (Fig. 8a). The proximal end is expanded anteroposteriorly, forming a posteriorly projected, mediolaterally compressed quadrangular flange (Fig. 8d), which has been suggested as a synapomorphy for Ornithomimosauria more derived than *Nqwebasaurus*^8^. The medial aspect of the proximal two-thirds of the diaphysis are occupied by a flat posteromedial surface, which forms a stout posterior ridge between the posteromedial and neighboring posterolateral planes (Fig. 8c), as seen in other ornithomimosaurs (e.g., Fig. 6 of Xu et al.^49^). The lateral surface forms a flat plane contacting metatarsal III in the proximal quarter (Fig. 8b). This flat plane distally becomes pinched to form a longitudinal ridge. In the distal half, another surface emerges dorsal to this ridge and expands anteriorly to form a broad plane facing anteromedially, also for the contact with metatarsal III (Fig. 8a). The distal end exhibits a single condyle in anterior view but is divided into a larger medial condyle and a smaller lateral condyle when seen from distal view (Fig. 8e) as in *Harpymimus*^27^. The intercondylar sulcus is deep, forming a distinct cleft as in other basal ornithomimosaurs such as *Harpymimus*, *Garudimimus* and *Aepyornithomimus* while the cleft is much shallower in more derived ornithomimosaurs^15, 22, 50, 51^.

The proximal part of metatarsal III is much narrower mediolaterally than anteroposteriorly and bears broad contact surfaces for the metatarsals II and IV on the medial and lateral aspects, respectively (Fig. 8f–h). These contact surfaces are expanded anteroposteriorly toward the proximal end to exhibit a triangular profile on each side above the slender diaphysis. In the proximal end, both contact surfaces approach with each other anteriorly to pinch the anterior surface of metatarsal III. The posterior aspect of the proximal part is occupied by the posterolateral surface and its proximal end is projected posterolaterally to form a rugose platform. The distal part of the diaphysis maintains the medial and lateral planes with both facing somewhat posteriorly and exhibits a medial expansion of the anterior surface (Fig. 8j–l). In combination with the broader anterior and narrower posterior surfaces, it exhibits a trapezoidal profile in the cross section (Fig. 8i). Whereas the anterior plane is confluent with the distal condyle, the posterior plane is pinched distally to disappear above the distal condyle (Fig. 8j, l). The distal condyle is not ginglymoid.

Metatarsal IV has a flat lateral surface forming a sharp longitudinal ridge on the posterolateral margin of the diaphysis (Fig. 8m) as seen in other ornithomimosaurs (Fig. 11 of Osborn^45^; Pl. 49 of Osmólska et al.^22^). The medial surface forms a flat plane contacting metatarsal III (Fig. 8n). This flat plane is anteroposteriorly expanded at the proximal end, pinched in the proximal half of the diaphysis, and again expanded in the distal half of the diaphysis. The distal part of the plane faces slightly more dorsally and forms a medial eminence for the articulation with metatarsal III (Fig. 8o). The distal part of the diaphysis bears a rugose surface along the posteromedial margin (Fig. 8m) that corresponds to the posteromedial ridge seen at least in one of the Eutaw ornithomimosaurs^52^.

#### Pedal phalanx

The presence of the first digit is indicated by pedal phalanx I-1 recovered as a part of the paratype (FPDM-V-10295; Fig. 8p, q). The proximal articular surface faces slightly medially and dorsally. The proximoventral margin is projected more proximally than the proximodorsal margin and the ventral surface bears a shallow depression. The lateral rim of this depression forms a blunt swelling on the lateroventral corner of the proximal part. The distal condyle is well developed as the ginglymus seen in penultimate phalanges of other digits. The articular surface faces distally and ventrally.

Pedal phalanx II-1 is represented by FPDM-V-11346 (Fig. 8r, s) and 11347. Pedal phalanx II-1 has a straight shaft with the lateral margin more concave than the medial margin in dorsal view. The proximal surface forms a single shallow concavity with an oval profile in proximal view, except for a concavity between a pair of ventral projections. These appears as a shallow groove and a pair of longitudinal ridges in ventral view. Medial to the medial ridge, the medioventral corner of the proximal shaft bears a distinct depression possibly for the insertion of m. interosseous dorsalis digiti II^53^. The distal condyle is ginglymoid and faces ventrally, distally and slightly dorsally. The lateral hemicondyle is slightly larger than the medial one. The lateral collateral ligament fossa is more deeply excavated and ventrally situated than the medial one as in other ornithomimosaurs. The hyperextensor pit is present on the dorsal surface just proximal to the distal condyle.

Pedal phalanx IV-1 and IV-2 are represented by FPDM-V-11348 and 11343 (Fig. 8t–w). The phalanges are proximodistally shorter than those of other digits as in other ornithomimosaurs. While the proximal surface of IV-1 bears a single concavity, the one of IV-2 bears two concavities divided by a vertical ridge inclined dorsolaterally. The ventral surface is flat below the proximal surface, and becomes convex in the diaphysis toward the ginglymoid distal condyle. The medial distal hemicondyle is much larger than the lateral one and reaches the mid-line at its dorsal margin as in other ornithomimosaurs. While the hyperextensor pit is present in both phalanges, it is indistinctive from the sulcus between the distal hemicondyles in IV-2 (Fig. 8w).

There are two pedal unguals in the paratype (FPDM-V-10295; Fig. 8x, y). The unguals are roughly triangular in dorsal, lateral and proximal views and have a straight ventral margin in lateral view. The ventral surface is mostly flat and proximally bears a pair of distinct pits and a slender flexor tubercle between them as in some ornithomimosaurs^54^.

## Discussion

Ornithomimosaur materials from the Bonebed 1 of the Kitadani Dinosaur Quarry seems to be monospecific based on the common appearance of features autapomorphic for the species within ornithomimosaurs. For example, in all dorsal neural arches referable to Ornithomimosauria, the spherical cavity is present at least within the prezygocentrodiapophyseal fossa. On the humerus, the deep anterolateral pit on the proximal part is present in all materials referable to Ornithomimosauria. Furthermore, although the distinct vertical ridge is not an autapomorphic feature in the group, it is present in all ilia preserving the corresponding part and referable to Theropoda to date. Although the unfused conditions of the braincase and the vertebral neurocentral sutures represent a possible immaturity of the holotype^55, 56^, the presence of diagnostic features seen in other known ornithomimosaurs supports that the holotypic individual is sufficiently matured to present ﻿apomorphies of *Tyrannomimus*. Similarly, the specimens described in the present study cannot be assigned to the juvenile of *Fukuiraptor* from the same stratigraphic layer because each of them presents a suite of characters present in ornithomimosaurs but absent in *Fukuiraptor*. For example, in *Tyrannomimus*, the dorsal centrum does not bear a deep lateral depression, the deltopectoral crest is not extended as a conical process, the manual ungual is not laterally compressed nor strongly recurved, the pubic peduncle of the ilium is not mediolaterally broad, the femur has a distinct trochanteric shelf, the fibular condyle of the tibia is relatively small and not clearly separated from the rest of the proximal end, and the metatarsals have broader and distinct articular surfaces to each other, unlike those of *Fukuiraptor*^57, 58^.

**Figure 9:**
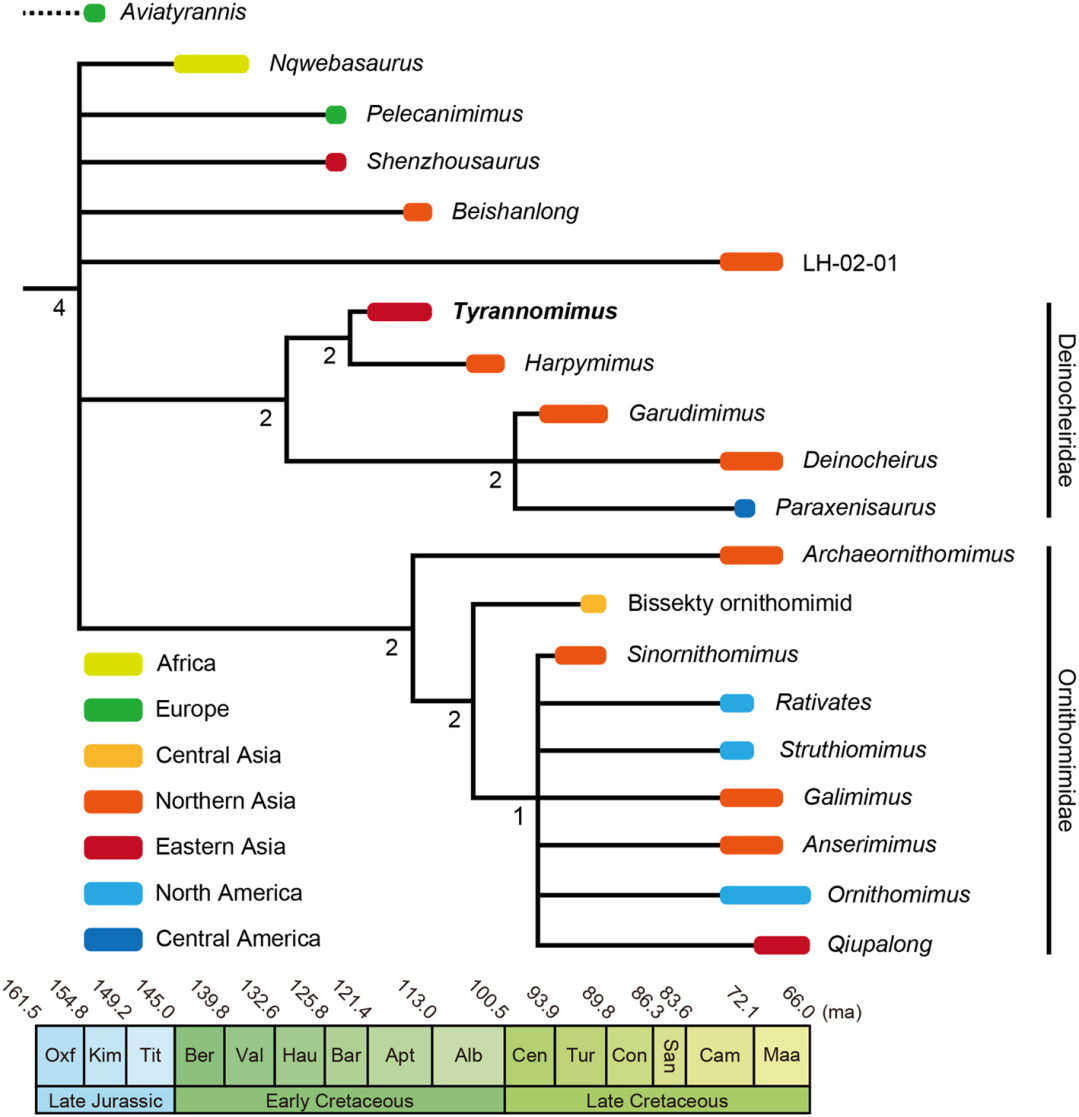
Phylogenetic position of *Tyrannomimus fukuiensis* within Ornithomimosauria. A colored bar in the strict consensus tree represents the time and region of occurrence for each taxon and a number represents Bremer support value for each node. Note that the dataset for the phylogenetic analysis that yielded this consensus tree did not include *Aviatyrannis*.

The phylogenetic analysis performed in the present study recovered 65 most parsimonious trees (MPTs) with a length of 3016 steps (consistency index equals 0.217; retention index equals 0.608). Additional Tree Bisection and Reconnection (TBR) branch swapping on these MPTs resulted in 2640 total MPTs. In the resulted strict consensus tree, *Tyrannomimus* is recovered as a sister taxon of *Harpymimus* within Deinocheiridae (Fig. 9; see Supplementary Fig. S1 online for the complete strict consensus). Although the analysis repeated with *Tyrannomimus* scored from the holotype alone collapsed the node of *Tyrannomimus* and *Harpymimus*, it does not remove *Tyrannomimus* from Deinocheiridae (see Supplementary Fig. S2 online).

The monophyly of Ornithomimosauria is supported by 44 unambiguous synapomorphies and *Tyrannomimus* presents 10 of them: (1) anteroposteriorly-elongated posterior dorsal vertebrae; (2) postzygapophyses of dorsal vertebrae abutting one another; (3) neural spines on posterior caudals forming low ridges; (4) shallow deltopectoral crest on humerus; (5) paired flexor processes on manual phalanges; (6) small flexor tubercles on manual unguals; (7) deeply concave brevis fossa on ilium; (8) ilium with process projecting into socket on ischium; (9) short phalanges of pedal digit IV; (10) straight pedal unguals. As a member of Deinocheiridae, *Tyrannomimus* has 4 of 11 unambiguous synapomorphies: (1) infracondylar fossa of the occipital condyle; (2) grooved ventral surfaces on anterior caudal vertebrae (convergent with *Archaeornithomimus*); (3) smooth lateral surface of the cnemial crest (convergent with most ornithomimids); (4) rectangular cross section of metatarsal III. The present analysis also recovered three autapomorphies of *Tyrannomimus* as follows: (1) deltopectoral crest shorter than the quarter of the humeral length (convergent with *Beishanlong*, *Gallimimus*, *Ornithomimus*, *Struthiomimus* and *Sinornithomimus*); (2) muscle scar on the anterolateral margin of the deltopectoral crest; (3) concave ventral margin of the postacetabular process (convergent with *Shenzhousaurus*).

The phylogenetic tree in this study indicates that *Tyrannomimus* constitutes the oldest member of Deinocheiridae and the first representative of the clade from eastern Asia (Fig. 9). Although the data is limited at present, it is notable that *Tyrannomimus* is recovered as a sister taxon to *Harpymimus* from the Albian of Mongolia^15, 59^, but not to geographically and temporarily proximate species *Shenzhousaurus* from the Barremian of northeastern China^30^. A similar case has been reported for hadrosauroid *Koshisaurus* from the Kitadani Formation^11^, where hadrosauroids are commonly present in the Early Cretaceous of Fukui and northern Asia but absent in northeastern China of the age. Additionally, the fossil cockroach assemblage from the Kitadani Formation is rather similar to the ones from the Early Cretaceous of Mongolia and Russia, than to the one from the Early Cretaceous of northeastern China^60^. Therefore, recovering *Tyrannomimus* as a sister taxon to *Harpymimus* provides an additional support for the hypothesis that the faunal composition of the Kitadani Formation has more in common with northern Asia than to northeastern China. Consequently, these studies including the present one may exemplify a case where it may be misleading to assume the faunal similarity simply based on the geographical and temporal proximities.

Because the ilium of *Tyrannomimus* is strikingly similar to the holotypic ilium of *Aviatyrannis* from the Upper Jurassic of Portugal, its purported affinity to a tyrannosauroid is tested here against an alternative hypothesis that it in fact belongs an ornithomimosaur. For example, the brevis fossa becomes mediolaterally broader to the posterior end in *Aviatyrannis*^38^ as in *Tyrannomimus* (Fig. 6) and other known ornithomimosaurs^7, 15, 30^, unlike the ones of tyrannosauroids with subparallel margins (e.g., Fig. 20 of Osborn^45^; Fig. 9 of Mallon et al.^46^). Although the well-developed vertical ridge on the lateral surface of the iliac blade had been known as a synapomorphy of Tyrannosauroidea^38^, it has been recognized in some ornithomimosaurs including *Tyrannomimus* (see Description for detail). Although the dorsal half of the anterior margin is concave in *Aviatyrannis*, it is much shallower than the notch seen in tyrannosauroids^61^. In addition, while some ornithomimosaurs lack the concavity on the anterior margin, many ornithomimosaurs do not have the corresponding portions preserved, so the conditions of these species remain unknown.

In addition to the direct comparisons above, the phylogenetic analysis was conducted with *Aviatyrannis* being included and scored based on the published description of the holotype^29^. The resulting tree suggests that *Aviatyrannis* is a sister taxon of *Tyrannomimus* without any other changes on the shape of the consensus trees from other analyses. This result leads to a hypothesis that *Aviatyrannis* represents not only the earliest ornithomimosaur but also the earliest deinocheirid (see Supplementary Fig. S3 online). On the other hand, when *Aviatyrannis* is included in the matrix in which *Tyrannomimus* is scored from the holotype alone, *Aviatyrannis* forms a node as a sister taxon of *Shenzhousaurus* outside Deinocheiridae and Ornithomimidae (see Supplementary Fig. S4 online). In any case, the ornithomimosaur affinity of *Aviatyrannis* appears plausible. Still, the position of *Aviatyrannis* within Ornithomimosauria remains ambiguous. While it is beyond the scope of the present study to reveal the phylogenetic position of *Aviatyrannis*, it is argued that the ornithomimosaur affinity of the genus needs to be tested further through the first-hand observation of the holotype and referred specimens.

The earliest definitive ornithomimosaur is known from the Lower Cretaceous (Berriasian–Valanginian) Kirkwood Formation of South Africa^24^ that is followed by several fragmentary specimens belonging to putative ornithomimosaurs *Valdoraptor* (Valanginian) from England^47^ and the Angeac theropod (Hauterivian–Barremian) from France^47^, as well as several fragmentary specimens belonging to *Kinnareemimus* (Hauterivian–Barremian) from Thailand^62^. Although another putative ornithomimosaur ﻿*Lepidocheirosaurus* is reported from the Upper Jurassic (?Tithonian) deposit of the Kulinda locality, Russia^63^, its affinity and identification of the taxon are problematic and the taxon is regarded as a nomen dubium in recent studies^64, 65^. Therefore, the recognition of *Aviatyrannis* from the Upper Jurassic (Kimmeridgian) Alcobaça Formation of Portugal^38^ as the earliest ornithomimosaur significantly expands the temporal range of Ornithomimosauria to the Kimmeridgian. It partially fills a 20-million-year ghost lineage of Ornithomimosauria implied by the presence of the earliest members of Maniraptora, the sister group of Ornithomimosauria, in the Middle Jurassic (Callovian)^66, 67^. In addition, the reevaluation of *Aviatyrannis* also expands the biogeographical range of early ornithomimosaurs to the Laurasia. This is consistent with the hypothesis that the biogeographical range of early ornithomimosaurs was widespread before the Pangaean breakup in the Kimmeridgian^47, 68^, while not eliminating another possibility that the dispersal between the Laurasia and Gondwana via the Early Cretaceous Apulian route^68, 69^ because both the route and *Nqwebasaurus* were present in the Early Cretaceous^24, 68^.

## Methods

Individual skeletal elements of *Tyrannomimus* were analyzed using a veterinary X-ray computed tomography (CT), Latheta LCT-200 (Hitachi, Ltd, Tokyo, Japan) with the following parameters: voltage of 80 kV, current of 500 µA, and voxel size of 0.048–0.080 mm (x- and y-axis) and 0.096–0.160 mm (z-axis) at Institute of Dinosaur Research, Fukui Prefectural University. FPDM-V-11333 was segmented from the acquired CT images and exported as a polygon mesh using Amira (v 2020.1, Thermo Fisher Scientific; Waltham, USA) while other specimens were rendered and exported using VGSTUDIO (v2.2, Volume Graphics; Heidelberg, Germany). Polygon meshes were then imported into Meshmixer (v3.5, Autodesk, San Rafael, USA) to observe, measure and capture images for figures under the orthographic projection view.

To assess the phylogenetic affinity of *Tyrannomimus*, type and referred specimens were scored into the latest version of the character-taxon matrix focused on the phylogeny of ornithomimosaurs^7^ that is derived from several previous studies^5, 6, 8, 9^. Some character descriptions and scores were modified based on information from literature. On character 440, the condition of *Shenzhousaurus* is modified from unknown to 1 due to the presence of the concave ventral margin of the postacetabular process^30^. On character 442, the conditions of *Shenzhousaurus* and *Harpymimus* are modified from state 0 to 2 due to the presence of the iliac vertical ridge in both taxa (see Description for detail). On character 444, the condition of *Shenzhousaurus* is modified from state 1 to 0 because the ischial peduncle forms a ventral conical process^30^. For character 557, scores are derived from Benson^70^, which cites character 51 of Gauthier^71^ and Currie and Zhao^72^. However, instead of the shape in the cross section, only the shape in the proximal surface of the metatarsus is discussed in both studies. Therefore, the description of this character should be modified from “Metatarsal III shape of shaft in cross section” to “Metatarsal III shape on proximal surface”. In *Nqwebasaurus*, the posterior margin is as wide as the anterior margin in proximal view (Fig. 23 of Sereno^65^), which is the primitive condition for this character^70, 72^ rather than the derived condition in character 557. In *Beishanlong*, metatarsal III forms an anteriorly-tapered wedge on the proximal surface^48^, in contrast to the derived condition of character 557.

The final dataset compiled with Mesquite 3.6^73^ includes 106 taxa covering an extensive sample of coelurosaurs and outgroups scored for 568 discrete anatomical characters. The following characters were ordered as in previous studies^5, 8^: 74, 82, 100, 119, 125, 132, 150, 179, 183, 219, 226, 227, 233, 236, 237, 263, 264, 272, 273, 278, 282, 302, 303, 311, 319, 322, 324, 325, 338, 347, 362, 366, 368, 371, 376, 405, 406, 410, 412, 417, 419, 421, 442, 444, 455, 479, 481, 494, 496, 533, 554 and 561. Following the previous studies^5–9^, the dataset was analyzed with equally weighted parsimony in TNT 1.5^74^ and *Herrerasaurus* was chosen as the outgroup to root the tree. At first, the dataset was analyzed under the “New Technology” search options, using sectorial search, ratchet, tree drift and tree fuse options with default settings, and the minimum tree length was found in 10 replicates to recover as many tree islands as possible. Subsequently, the results were exposed to the branch-swapping algorithm of Tree Bisection Reconnection (TBR). The supporting values for each node were calculated as Bremer support (with retaining trees suboptimal by five steps) and bootstrapping score (with 10,000 replicates).

### Nomenclatural acts

This published work and the nomenclatural acts it contains have been registered in ZooBank, the proposed online registration system for the International Code of Zoological Nomenclature (ICZN). The ZooBank LSIDs (Life Science Identifiers) can be resolved and the associated information viewed through any standard web browser by appending the LSIDs to the prefix http://zoobank.org/. The LSIDs are 0CF940C0-EE3C-4509-B939-60664B66CB0B for this publication, BE398021-AB04-44C9-828F-3E234C2F59EC for the new genus *Tyrannomimus*, and 17A65040-FA55-493E-9740-4D693F5501E0 for the new species* T*. *fukuiensis*.

### Supplementary Information

Supplementary Figures.

Supplementary Information.

## Data Availability

The specimens described here are housed at public and permanent repository in the collection of Fukui Prefectural Dinosaur Museum and accessible to all researchers. All relevant data including a phylogenetic dataset are within the manuscript and its Supplementary Information.

## Abbreviations

AMNH: American Museum of Natural History, New York, USA
FPDM: Fukui Prefectural Dinosaur Museum, Fukui, Japan
LH: Long Hao Institute of Geology and Paleontology, Hohhot, Nei Mongol Autonomous Region, China
UCMP: University of California Museum of Paleontology, Berkeley, USA
