# Author Correction: New theropod dinosaur from the Lower Cretaceous of Japan provides critical implications for the early evolution of ornithomimosaurs

**DOI:** 10.1038/s41598-023-43487-y

**Published:** 2023-09-28

**Authors:** Soki Hattori, Masateru Shibata, Soichiro Kawabe, Takuya Imai, Hiroshi Nishi, Yoichi Azuma

**Affiliations:** 1 Institute of Dinosaur Research, Fukui Prefectural University, 4‑1‑1 Matsuoka‑kenjojima, Eiheiji, Fukui 910‑1195 Japan; 2 Fukui Prefectural Dinosaur Museum, 51‑11, Terao, Muroko, Katsuyama, Fukui 911‑8601 Japan

Correction to: *Scientific Reports* 10.1038/s41598-023-40804-3, published online 07 September 2023

The original version of this article contained an error in Supplementary Information file 1, where Figure S2 was a duplication of Figure S3.

The original Article and accompanying Supplementary Information file have been corrected.

