# Supplementary material for: New theropod dinosaur from the Lower Cretaceous of Japan provides critical implications for the early evolution of ornithomimosaurs
Source: Sci Rep. 2023 Sep 7;13:13842. doi: 10.1038/s41598-023-40804-3 (PMC10484975; doi:10.1038/s41598-023-40804-3)
Supplement: Supplementary file 1 — Supplementary Figures. [file 41598_2023_40804_MOESM1_ESM.pdf]

Supplementary Information

New theropod dinosaur from the Lower Cretaceous of Japan provides critical implications for the early evolution of ornithomimosaurs

Soki Hattori, Masateru Shibata, Soichiro Kawabe, Takuya Imai, Hiroshi Nishi, Yoichi Azuma

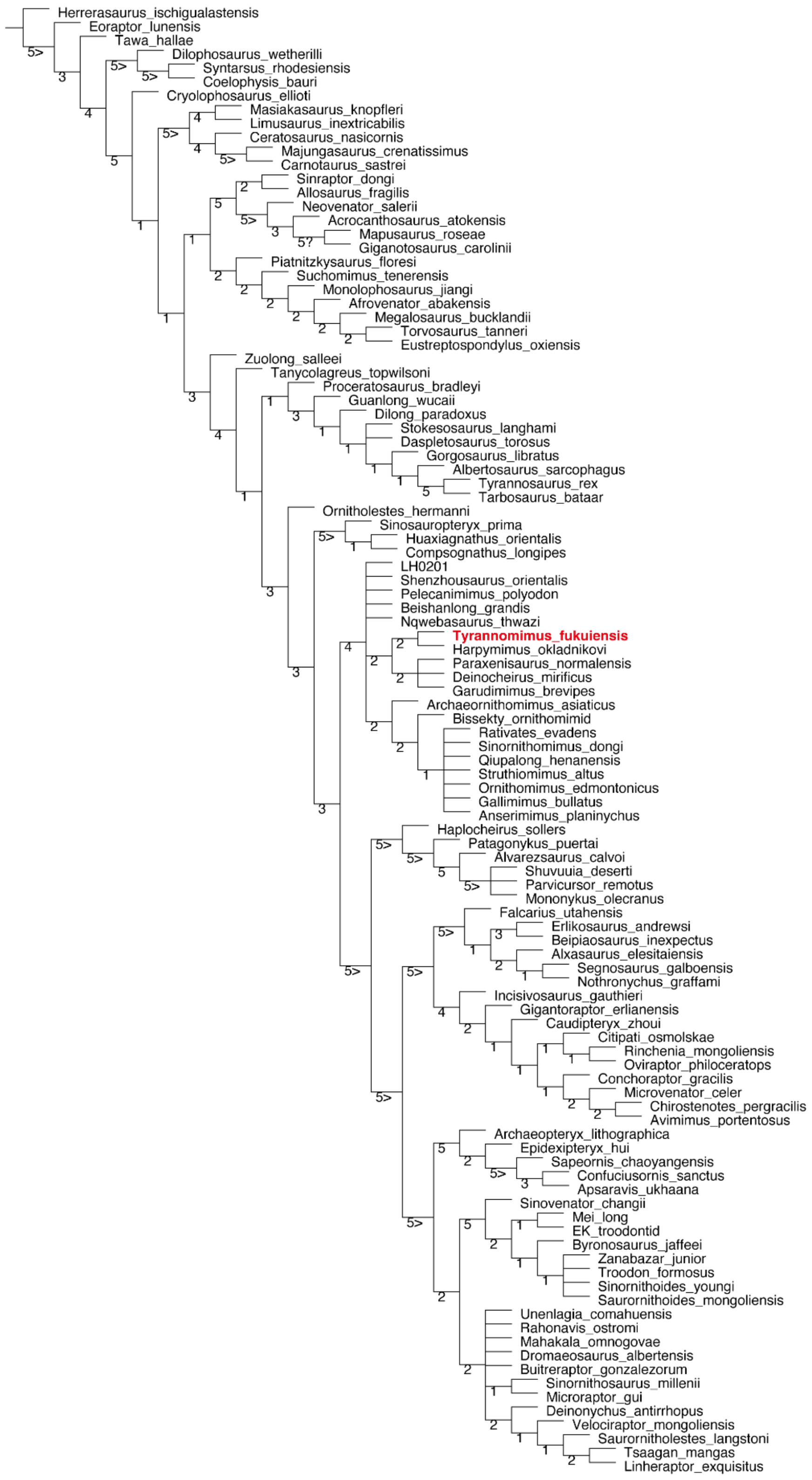

Figure S1. Strict consensus tree of 2640 MPTs with a length of 3016 steps (CI = 0.217; RI = 0.608) resulted by the phylogenetic analysis conducted in the present study. Numbers associated with nodes represent Bremer support values.

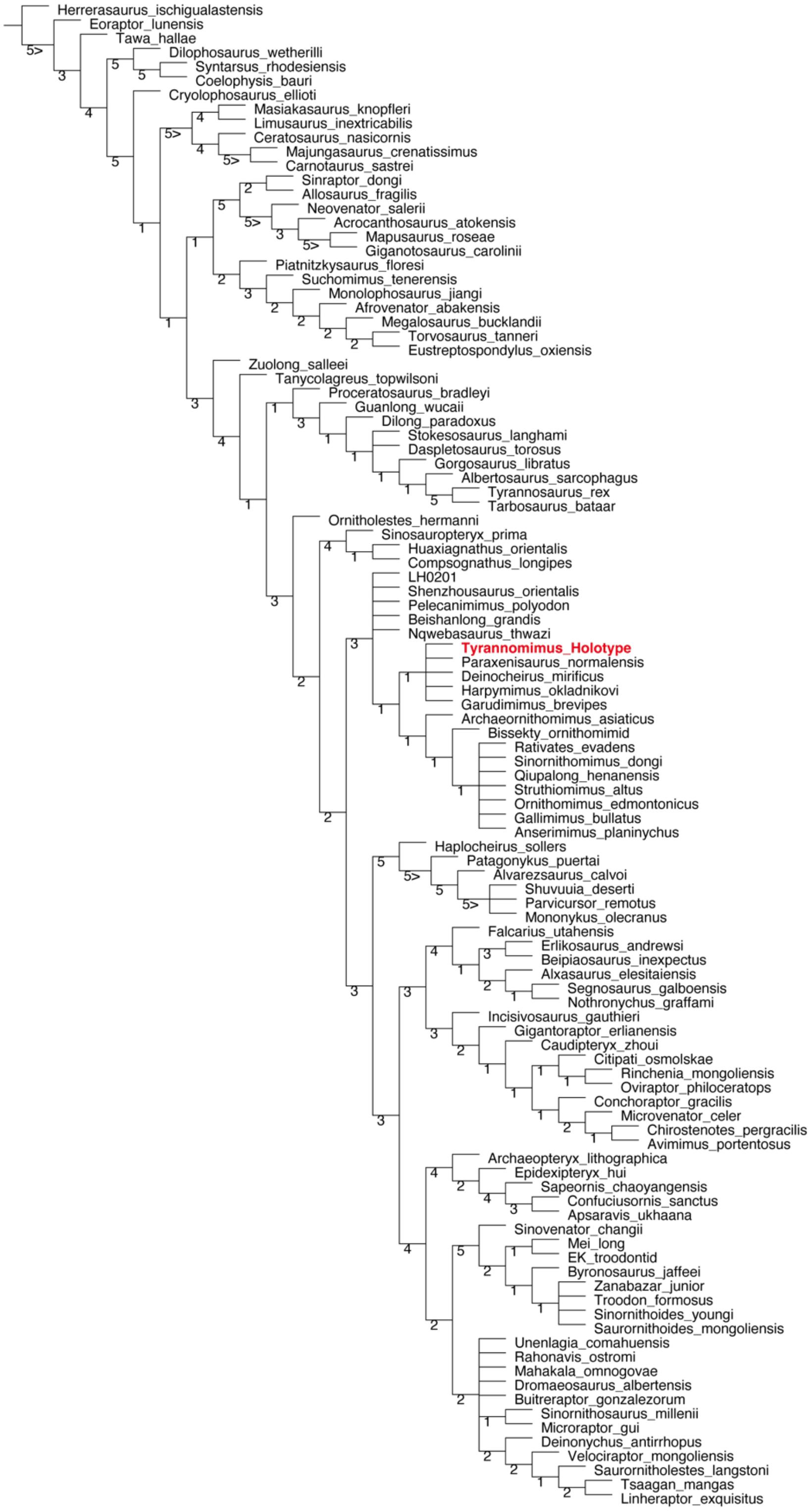

Figure S2. Strict consensus tree of 5280 MPTs with a length of 3007 steps (CI = 0.219; RI = 0.610) resulted by the phylogenetic analysis conducted with *Tyrannomimus fukuiensis* scored from the holotype alone. Numbers associated with nodes represent Bremer support values.

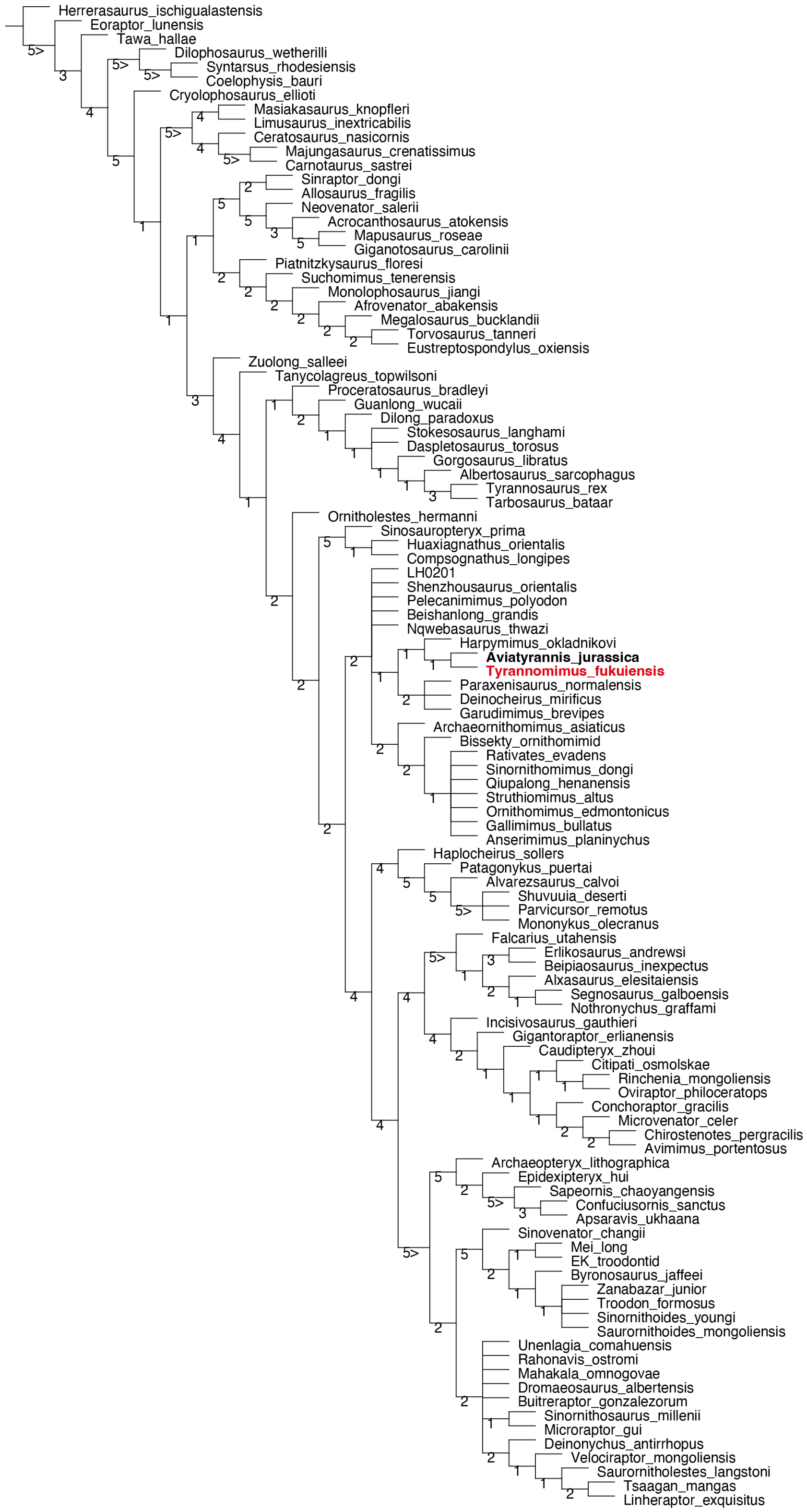

Figure S3. Strict consensus tree of 2640 MPTs with a length of 3019 steps (CI = 0.217; RI = 0.608) resulted by the phylogenetic analysis conducted with *Aviatyrannis jurassica* being added. Numbers associated with nodes represent Bremer support values.

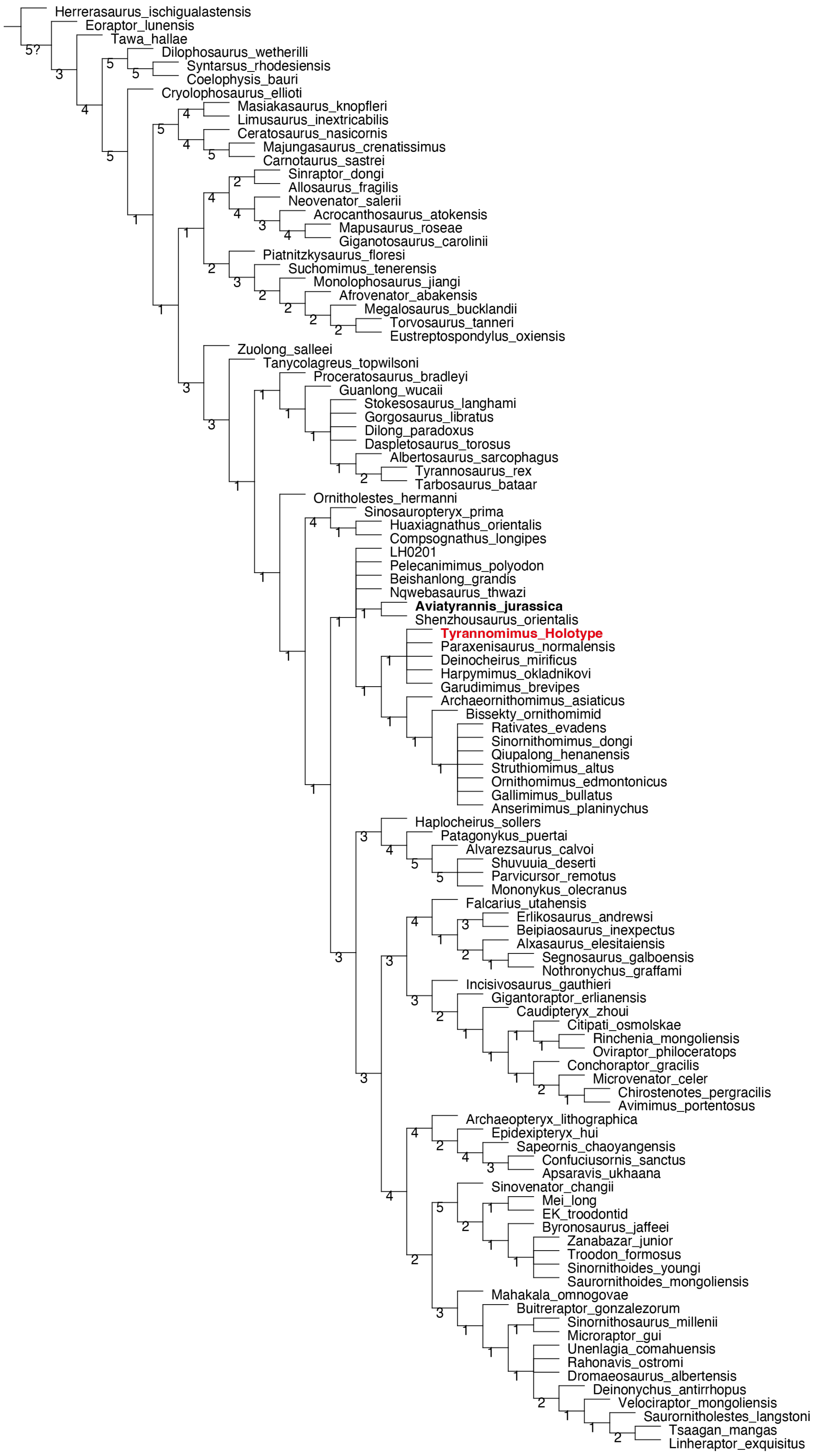

Figure S4. Strict consensus tree of 7920 MPTs with a length of 3011 steps (CI = 0.216; RI = 0.606) resulted by the analysis conducted with *Tyrannomimus fukuiensis* scored from the holotype alone and *Aviatyrannis jurassica* being added. Numbers associated with nodes represent Bremer support values.
